# Progress of alveolar macrophages in biological function and acute lung injury/acute respiratory distress syndrome

**DOI:** 10.3389/fimmu.2025.1683411

**Published:** 2025-11-27

**Authors:** Mi Yan, Jia Tang, Yanfei Liu, Zhangxue Hu

**Affiliations:** Department of Pediatrics, Daping Hospital, Army Medical University, Chongqing, China

**Keywords:** acute lung injury, acute respiratory distress syndrome, macrophages, cell polarization, cell death

## Abstract

Alveolar macrophages (AMs), a type of immune cell, display remarkable plasticity and manifest diverse responses to stimuli by differentiating into distinct subgroups. These phenotypically distinct macrophage subtypes are primarily categorized as either classically activated or inflammatory (M1) macrophages, or alternatively activated or anti-inflammatory (M2) macrophages, the differentiation of which is underpinned by a complex regulatory network. Despite their crucial contribution to the pathobiology of acute lung injury/acute respiratory distress syndrome (ALI/ARDS), the research on AMs is currently limited. This study therefore aims to establish a comprehensive theoretical framework delineating the pathogenic role of AMs in ALI/ARDS, facilitating deeper mechanistic understanding of disease initiation and progression in ALI/ARDS and ultimately identifying novel therapeutic targets.

## 1 Introduction

Acute lung injury (ALI) is a prevalent and consequential medical condition that poses a substantial risk to human health outcomes (1). This definition characterizes the condition as a rapid onset of inflammation in the lungs that is caused either directly by injury to the lungs or indirectly by extrapulmonary processes. Such inflammation can ultimately result in qualitative or quantitative surfactant dysfunction, thus leading to further complications. The primary features of acute respiratory distress syndrome (ARDS) include varying degrees of hypoxemia, diffuse bilateral lung transmittance reduction, inflammatory exudation, and decreased lung compliance (2). Despite extensive research, medication alone cannot provide specific treatment for ALI/ARDS. The primary form of treatment is supportive therapy, which includes extracorporeal membrane oxygenation, mechanical ventilation, liquid therapy, and prone position ventilation. Although the clinical outcomes of ALI/ARDS patients have improved with the use of these therapies, the mortality rate remains significant (3). Therefore, identifying efficient treatment targets is essential. ARDS is a severe consequence of ALI, and ongoing inflammation of the airways or alveoli characterizes the condition. Among the various immune cells involved in the pathology of ALI, Alveolar macrophages (AMs) play a pivotal role (4). AMs are indispensable constituents of the immune system and exhibit pivotal functions in the pathobiology of diverse acute disorders (5).

AMs are long-lived, tissue-resident macrophages in the airways and alveoli that exhibit minimal turnover under homeostatic conditions (6). As the principal immune sentinels of the respiratory tract, AMs possess distinct phenotypic and transcriptional signatures that enable them to regulate pulmonary inflammation and maintain immune equilibrium. Under physiological conditions, AMs remain relatively quiescent; however, upon exposure to pathogens or inflammatory stimuli, they are rapidly activated through pattern-recognition receptors, leading to the release of cytokines and chemokines that facilitate inflammation resolution and tissue repair. Impaired efferocytosis and aberrant polarization of AMs have been implicated in the pathogenesis of ARDS. For instance, IL-8 has been shown to promote classical (M1) macrophage activation, while its inhibition enhances apoptotic cell clearance and mitigates inflammation (7). Macrophage polarization is dynamically shaped by local microenvironmental cues, resulting in the differentiation into either classically activated M1 or alternatively activated M2 phenotypes. M1 macrophages predominantly drive pro-inflammatory responses, whereas M2 macrophages orchestrate anti-inflammatory and reparative processes (8). The M2 phenotype can be further subdivided into four subtypes—M2a, M2b, M2c, and M2d—each exerting unique regulatory effects during inflammation and tissue remodeling (9). In the context of ALI, M1 macrophages contribute primarily to the early inflammatory phase, while M2 macrophages are more involved in the resolution and repair stages (8). Each macrophage subtype performs specialized functions that collectively maintain immune equilibrium; therefore, achieving an appropriate balance between M1 and M2 polarization is critical for the prevention and resolution of ALI/ARDS.

Moreover, AMs serve as crucial regulators of pulmonary immune homeostasis by modulating the activity of other immune cells. Depletion of AMs has been shown to amplify inflammatory responses in experimental models (7). Through the secretion of pro-inflammatory cytokines and chemokines, macrophages recruit neutrophils and other leukocytes to initiate and coordinate innate immune defense. Beyond their immunoregulatory role, macrophages are indispensable for maintaining tissue integrity and facilitating repair following injury. Their diverse functions include the clearance of apoptotic cells, necrotic debris, and pathogens; direct crosstalk with alveolar epithelial cells; and the secretion of growth factors that promote epithelial regeneration and tissue remodeling. Owing to their remarkable plasticity, macrophages exhibit dynamic phenotypic and functional adaptations in response to the local microenvironment (3). This versatility enables them to transition between pro-inflammatory and reparative states depending on contextual cues. In addition, macrophages actively participate in extracellular matrix remodeling, angiogenesis, and the resolution of inflammation, processes essential for restoring lung structure and function (8).

Numerous studies have demonstrated that AM-mediated cell death occurs across various models of ALI, suggesting that targeting these regulated cell death (RCD) pathways may represent a promising therapeutic strategy for ALI and ARDS (7). Cell death is a fundamental biological process essential for maintaining tissue homeostasis, development, and regeneration, as well as for eliminating damaged or potentially malignant cells. However, excessive or dysregulated RCD can provoke systemic inflammation and lead to pathological tissue damage. As key effectors of the pulmonary immune response, AMs not only serve as the first line of defense against pathogens and participate in the clearance of apoptotic and necrotic cells but also contribute to tissue repair and immune regulation (7). In the context of ALI/ARDS, AMs have been reported to undergo multiple forms of cell death, including apoptosis, autophagy-dependent cell death, pyroptosis, ferroptosis, and necroptosis (7, 10–13). Despite growing evidence, the regulatory mechanisms governing these processes remain incompletely understood. This review therefore aims to summarize the origin, polarization phenotypes, immune regulatory functions, metabolic characteristics, and death modalities of alveolar macrophages, as well as their involvement in the pathogenesis of ALI/ARDS. The insights discussed herein may provide a theoretical basis for the development of novel therapeutic approaches targeting AMs in these inflammatory lung disorders.

## 2 Origin of alveolar macrophages

Macrophages are immune cells widely distributed in the blood and body tissues. Together with peripheral blood monocytes and dendritic cells, they belong to the mononuclear phagocyte system and play crucial roles in immune defense, surveillance, and homeostasis (14, 15). In vertebrates, there exist at least three types of hematopoietic progenitor cells: primitive hematopoietic progenitors, erythro-myeloid progenitors, and hematopoietic stem cells, which possess distinct genetic backgrounds and developmental processes (16, 17). Current consensus delineates distinct ontogenic pathways: monocyte-derived macrophages emerge through bone marrow definitive hematopoiesis, while tissue macrophage progenitors originate from yolk sac and fetal liver via primitive/definitive hematopoiesis. Macrophages derived from tissue-resident cells had distinct morphologies, gene expression patterns, and functional properties compared to monocytes recruited from circulation to sites of inflammation. Notably, embryonic-origin macrophages preserve self-renewal capacity, contrasting with terminally differentiated monocyte-derived populations (18).

**Figure 1:**
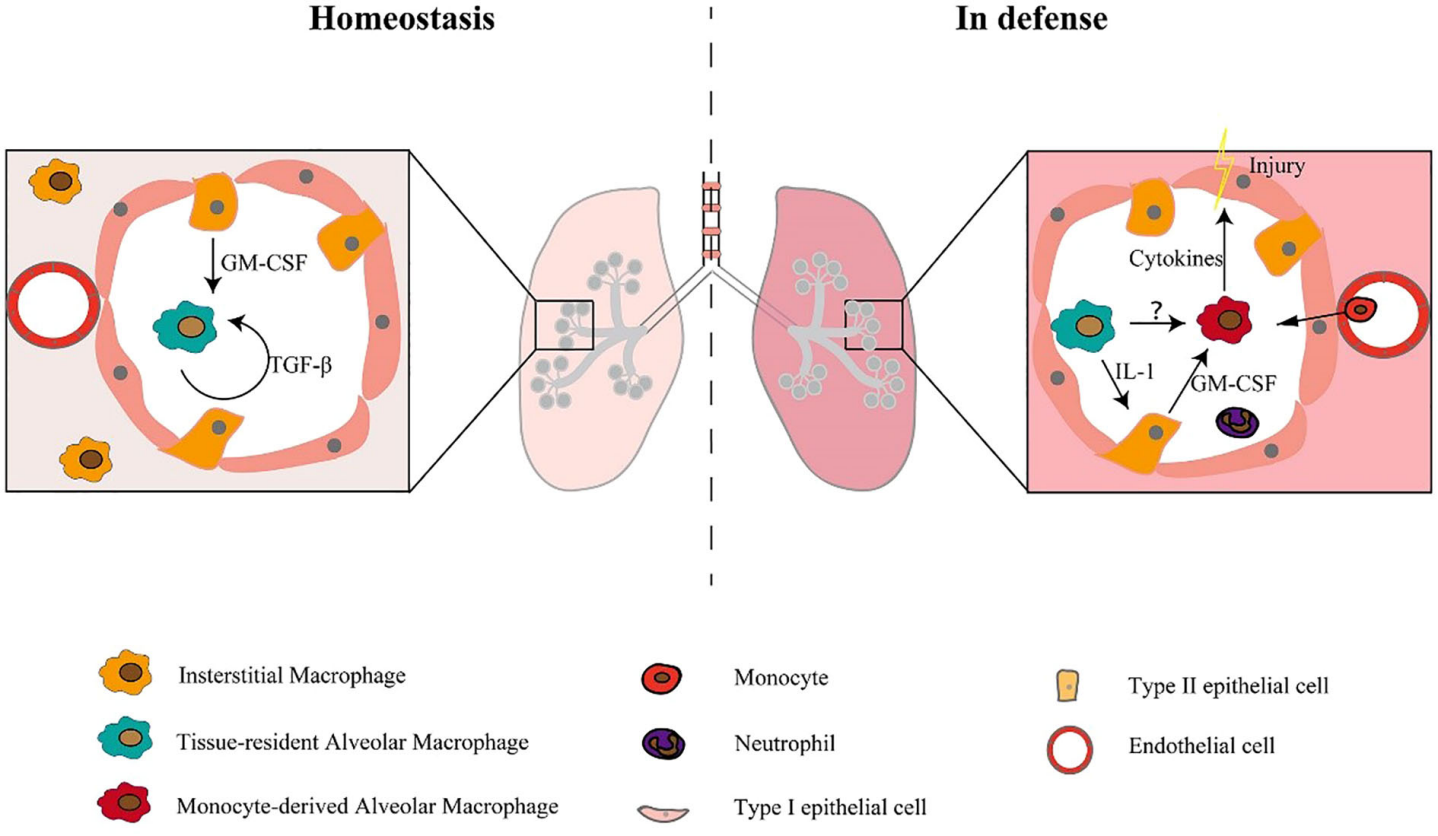
Macrophage subpopulations exist in both stable and defensive states (5).

Pulmonary macrophages are anatomically divided into three categories: AMs, pulmonary interstitial macrophages (PIMs), and pulmonary vascular macrophages (PVMs). These macrophage populations are localized in distinct anatomical areas within the lung (3). These macrophages form a complex network within the pulmonary compartments. The most abundant type of pulmonary macrophages are AMs, which are found in the alveolar spaces. Their primary function is to phagocytose and clear inhaled particles, such as bacteria, viruses, and debris. On the other hand, PIMs are located in the lung interstitium, where they control immune responses and play a part in tissue repair and remodeling. PVMs live in the walls of pulmonary blood vessels and take part in immune surveillance and clearance of intravascular pathogens. The functions of these macrophage subpopulations are specialized and cooperative, contributing to the maintenance of normal respiratory function and host defense in the lungs (5). In the pulmonary system, two primary macrophage classifications, i.e., AMs and interstitial macrophages (IMs). AMs and IMs are considered tissue-resident macrophages (TRMs) and have essential functions in maintaining homeostasis, metabolism, and repair required by organs, while also acting as sentinels for phagocytosis of immune cells. However, there are significant differences in the transcription profile, ontogeny, phenotype, location, and function of lung TRMs (19). AMs and IMs display distinct characteristics in both the normal physiological state and in pathological conditions (5). AMs are the first line defenders of alveoli and airways, while pulmonary IMs act as gatekeepers of the vascular system and pulmonary interstitium (19). AMs display unique origin and characteristics, which enable their classification into two distinct categories: tissue-resident alveolar macrophages (TR-AMs) and monocyte-derived alveolar macrophages (Mo-AMs). IMs were initially discovered in the pulmonary interstitium, while AMs were found in the alveolar area (20, 21). Monocytes possess the unique capacity to differentiate into two distinct forms of AMs, namely TR-AMs and Mo-AMs. TR-AMs originate from yolk sac hematopoiesis during embryonic development and sustain themselves through local proliferation, while Mo-AMs derive from circulating blood monocytes that penetrate the pulmonary tissue following an inciting event, such as injury or infection (as shown in **Figure 1**) (5). Mo-AMs display distinct phenotypic and metabolic characteristics from TR-AMs, and are more susceptible to alterations by the lung microenvironment (22, 23). Additionally, the ripening and self-maintenance of AMs is dependent on factors including GM-CSF and TGF-β (as illustrated in **Figure 1**). In mice, lacking GM-CSF may affect AM development, rendering them more vulnerable to disease-causing agents (5). In all, AMs, representing canonical tissue-resident populations, functionally gatekeep pulmonary homeostasis and drive post-damage tissue repair in the context of ALI/ARDS.

Under steady-state conditions, the maturation and self-sustainability of tissue-resident alveolar macrophages are dependent on GM-CSF and TGF-β. Under defense-state conditions, monocytes recruit into the alveolar cavity and differentiate into monocyte-derived alveolar macrophages, which release cytokines and lead to tissue damage (5).

## 3 Macrophage polarization and cytokines

Macrophages of distinct cellular origins exhibit divergent polarization phenotypes, which consequently execute specialized functions. The phenomenon of macrophage polarization is a unique and noteworthy feature whereby these cells are endowed with the ability to rapidly generate a distinct phenotype and functional response when exposed to varying pathophysiological circumstances and their microenvironment. This plasticity allows macrophages to respond to a wide range of stimuli and adapt to different stages of disease progression (24, 25). The development of ALI/ARDS is largely influenced by the response of severe inflammation. Approximately 90% of the white blood cells in the air space are AMs, which are situated near the air-tissue interface (9). Amultitude of academic research studies have consistently demonstrated the pivotal role played by macrophages, comprising both locally resident AMs and circulating monocyte-derived macrophages, in the pathogenesis and progression of ALI and ARDS (5, 9). It is worth highlighting that macrophages can adopt either pro-inflammatory (M1) or anti-inflammatory (M2) phenotypes depending on the specific pathophysiological context (8). In the normal state, the predominant polarized phenotype of resident AMs is the M2 phenotype, which represents a homogeneous, static, and immunosuppressive population. However, in response to pathogenic stimuli such, resident AMs can switch to the M1 phenotype and produce inducible NO and TNF-α at significant levels (3). On the other hand, M2 macrophages are stimulated by anti-inflammatory cytokines and function to suppress the immune response by generating anti-inflammatory cytokines, repairing damaged tissue, and encouraging tissue regeneration (26). Despite their beneficial roles, excessive polarization towards either M1 or M2 phenotypes can be detrimental. Excessive M1 polarization can result in uncontrolled inflammation and tissue damage, while skewed M2 polarization can lead to suppressed inflammation. As ARDS is a complex inflammatory condition involving the dysregulation of immune responses, balancing the M1/M2 macrophage ratio could indeed be a potential therapeutic strategy.

**Figure 2:**
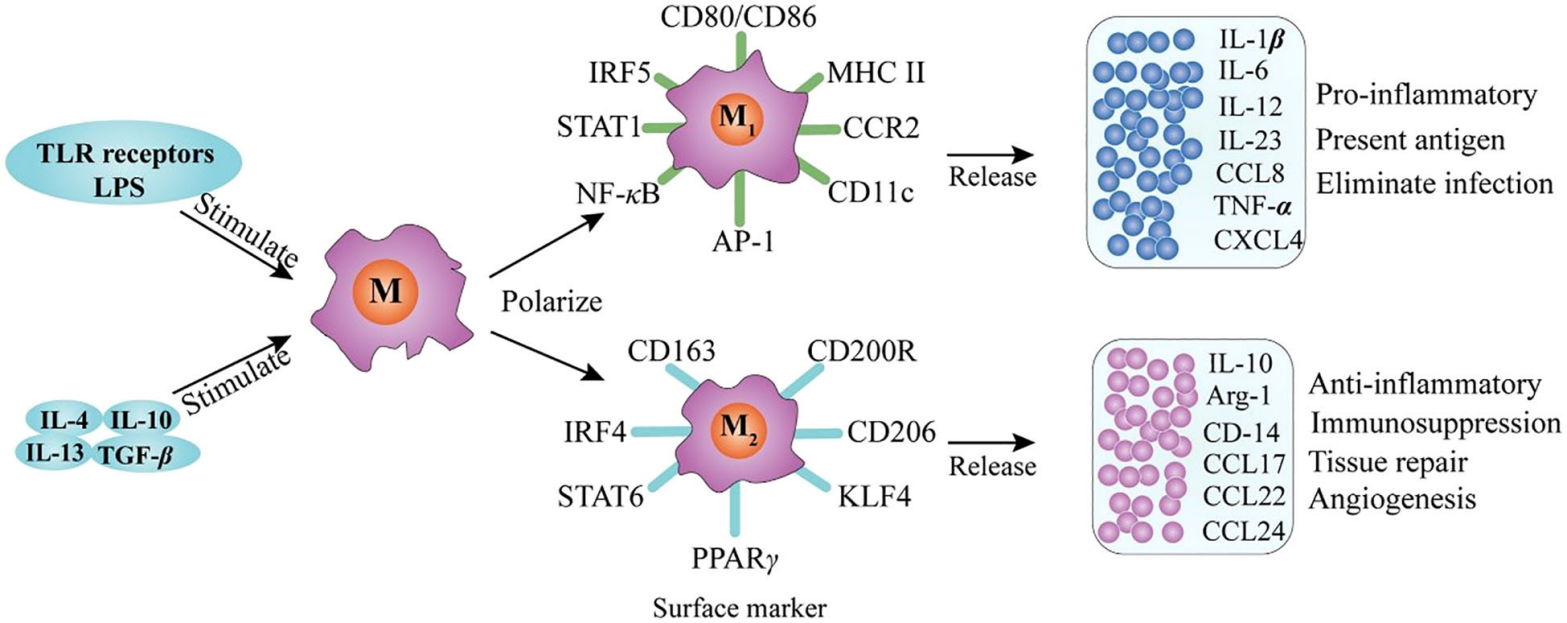
Macrophage polarization and phenotype (27).

**Figure 3:**
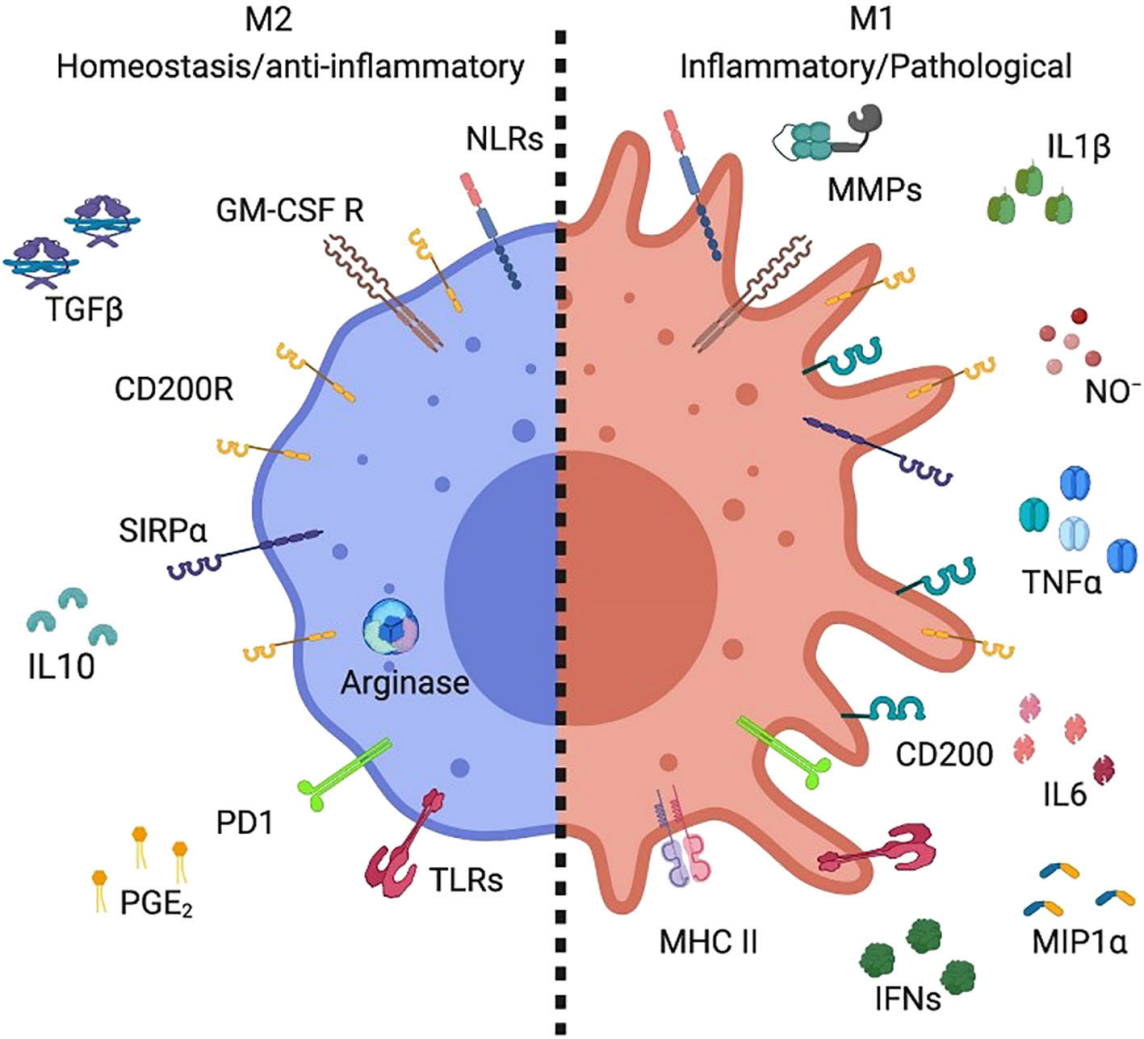
A specific subset of alveolar macrophages produced distinct markers (28).

Macrophage polarization into M1 and M2 phenotypes is primarily governed by gene expression–dependent mechanisms that regulate cell surface marker expression, cytokine secretion profiles, and metabolic reprogramming. Typically, M1 macrophages exhibit a pro-inflammatory phenotype, whereas M2 macrophages display anti-inflammatory and tissue-repairing properties (25). M1 polarization is driven by stimuli such as lipopolysaccharide (LPS) and Toll-like receptor (TLR) activation, which induce the production of pro-inflammatory cytokines—including interleukin-1β (IL-1β), IL-23, IL-12, IL-6, and tumor necrosis factor-α (TNF-α)—as well as chemokines such as CXCL2, CCL8, CXCL4, and CCL5 (shown in **Figure 2**) (17). Conversely, M2 macrophages comprise multiple subsets that respond to distinct immunological cues. M2a macrophages are induced by interleukin-4 (IL-4) and interleukin-13 (IL-13); M2b macrophages are activated by immune complexes in combination with LPS or IL-1β; and M2c macrophages are stimulated by anti-inflammatory mediators such as glucocorticoids, IL-10, or transforming growth factor-β (TGF-β). All M2 subsets express CD14 and produce abundant anti-inflammatory molecules, including IL-10, arginase-1, CCL24, CCL22, and CCL17 (17). As illustrated in **Figure 3**, M2 macrophages also express GM-CSFR, CD200R, and SIRP1α (27). M1-type AMs, which are associated with inflammatory pathology, produce mediators such as matrix metalloproteinases, nitric oxide (NO), IL-1, interferons, IL-6, TNF, and macrophage inflammatory protein-1, and upregulate CD200 and MHC class II expression (**Figure 3**). In contrast, M2-type AMs, which are considered more stable and anti-inflammatory, express high levels of arginase and secrete IL-10, prostaglandin E2, and TGF-β (**Figure 3**) (28). Although the dichotomy between M1 and M2 macrophages provides a valuable framework for understanding macrophage biology, it may not fully capture the phenotypic and functional continuum exhibited by AM populations *in vivo* (24).

The polarization and phenotype of macrophages have been extensively studied and are known to be modulated by a myriad of key factors. These factors include TLR receptor signal transduction and exposure to various stimulatory agents such as interleukins IL-4, IL-10, and IL-13, as well as transforming growth factor beta (TGF-β) (27).

M2 AMs exhibited high levels of arginase expression along with the production of interleukin-10 (IL-10), prostaglandin E2 (PGE2), and transforming growth factorbeta (TGF-b). In contrast, M1 Ams produced inflammatory mediators such as metallopeptidase, nitric oxide (NO), interleukin-1 (IL-1), interferon (IFN), interleukin-6 (IL-6), tumor necrosis factor (TNF), and macrophage inflammatory protein-1 (MIP-1) (28).

AMs are indispensable components of the pulmonary immune system, exhibiting diverse functions and phenotypic traits that depend on their activation state (24). Similar to other tissue-resident macrophages, the functional heterogeneity and adaptability of AMs are largely shaped by their surrounding microenvironment (29, 30). The differentiation of tissue macrophages parallels that of helper T cells, with M1 macrophages representing the classically activated, pro-inflammatory phenotype and M2 macrophages constituting the alternatively activated, anti-inflammatory counterpart (31). M1 macrophages possess enhanced antigen-presenting capacity and a robust pro-inflammatory profile, playing a critical role in host defense against bacterial, viral, and fungal pathogens (17). In contrast, M2 macrophages are essential for the resolution of inflammation, as they suppress excessive immune activation and promote wound healing, tissue repair, and angiogenesis (24, 28). However, their antimicrobial capacity remains limited. It is important to note that these macrophage phenotypes are primarily defined based on *in vitro* activation models, and further investigation is required to determine how accurately these categories reflect *in vivo* macrophage states and functions.

### 3.1 Epigenetics regulates macrophage polarization

During the differentiation and activation of macrophages, there was a noticeable epigenetic remodeling that occurred to adjust to changes in their transcription pool (32). The polarization of M1 and M2 macrophages results in distinct transcriptional processes. Furthermore, a growing body of experimental research has indicated that epigenetic mechanisms, such as histone acetylation and methylation, play a role in controlling the transcriptional diversity between the two different subsets of macrophages (33). DNA methylation is a vital epigenetic modification that has a significant impact on various regulatory mechanisms involved in life processes, cell differentiation, and development (34). The methylation status of a gene promoter is a reliable indication of gene activity and can establish a link between epigenetic modifications, gene expression, and protein levels. While DNA methylation is a crucial epigenetic modification involved in regulating macrophage polarization (35), there is still much to learn about the other regulatory mechanisms at play. Additionally, more high-throughput methods are needed to validate the functional consequences of newly discovered genes associated with macrophage polarization. Overcoming these limitations has the potential to provide further understanding of the intricacies involved in macrophage polarization and may reveal novel targets that could be pursued for the treatment of related diseases.

Macrophage polarization is also regulated by several miRNAs and long noncoding RNAs (lncRNAs). Several miRNAs, including miR-375, miR-let7, miR-34a, miR-155, miR-124, miR-511-3p, miR-99a, miR-132, and miR-145-3p have been identified as regulators of macrophage polarization. In addition, certain lncRNAs also affect macrophage polarization. This biological process is crucial in the development of several human diseases, especially those linked to immune system dysfunction and cancer formation, emphasizing its fundamental role in biological function (36). lncRNAs are a type of RNA molecule with a minimum length of 200 nucleotides. While structurally resembling messenger RNA (mRNA), lncRNAs do not have the conventional capacity to encode proteins (35). Presently, lncRNAs are classified into various categories, including long intergenic ncRNAs, natural antisense transcripts, transcripts with unclear coding potential, enhancer RNAs, and lncRNAs derived from pseudogenes (37).

**Figure 4:**
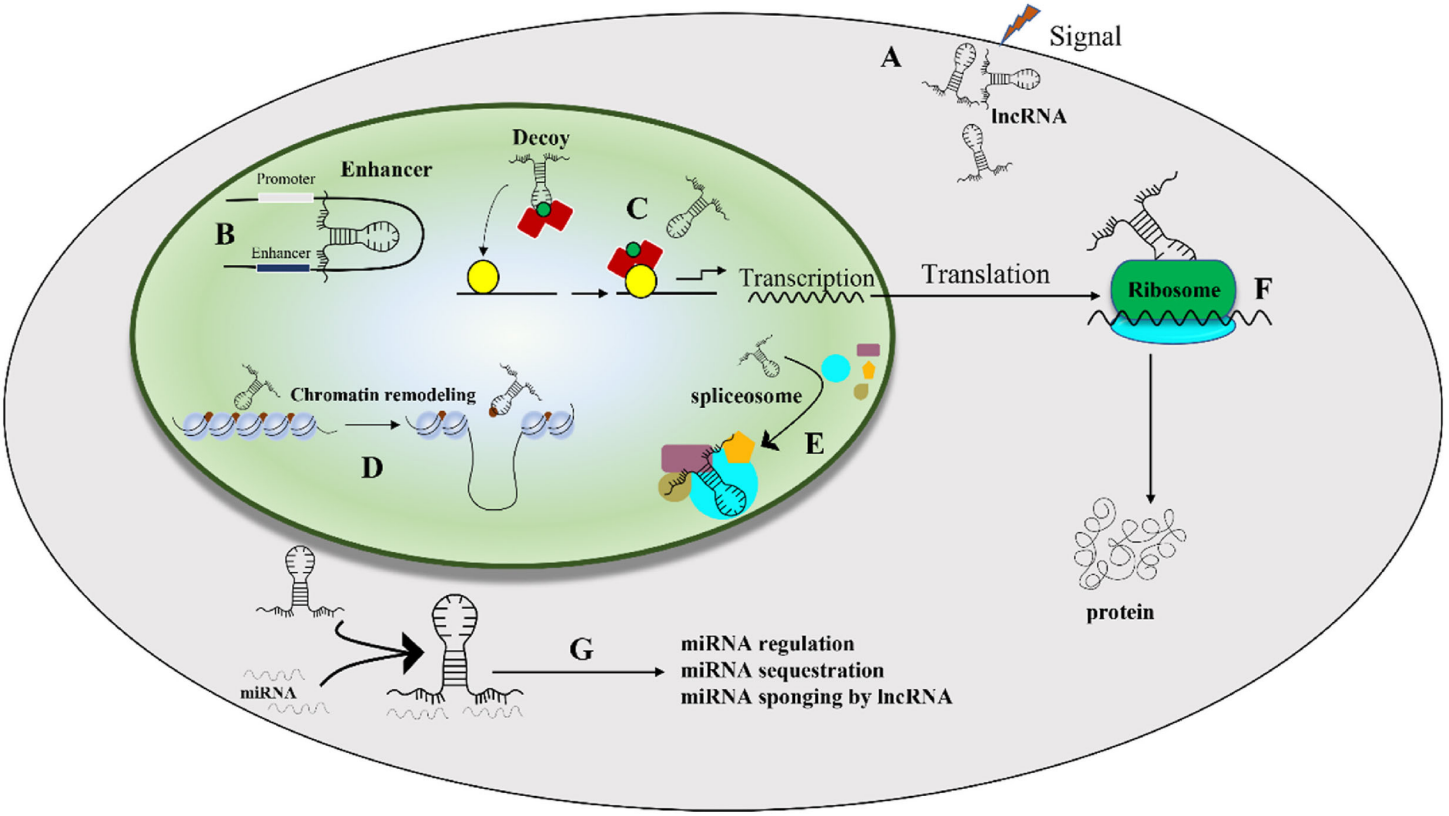
The situation of lncRNA activity in the regulation of cell activity (38). **(A)** lncRNAs respond to external stimuli by altering their expression levels, thereby influencing downstream cellular processes; **(B)** they regulate promoter and enhancer regions of genes, which in turn modulates gene expression, and **(C)** lncRNAs provide a platform for transcriptional activity by interacting with transcription factors and RNA polymerase. **(D)** lncRNAs control chromatin remodeling by recruiting chromatin-modifying enzymes; **(E)** They are involved in splicing alteration through their interactions with splicing bodies. **(F)** lncRNAs modulate translation and protein modification, which further affects cellular function, and **(G)** microRNAs can regulate lncRNAs, which in turn can influence various cellular activities (38).

LncRNAs have emerged as a crucial regulator of nuclear transport, chromatin remodeling, and other biological processes (as shown in **Figure 4**) (37, 38). In empirical investigations, it has been observed that lncRNAs serve as important regulators of gene expression through their interactions with epigenetic regulatory factors, resulting in significant alterations in various aspects of chromatin architecture. Extensive research has confirmed the contribution of lncRNAs in numerous human pathologies. Furthermore, recent studies have elucidated the underlying mechanisms by which lncRNAs participate in macrophage differentiation and activation pathways (35). Numerous investigations have underscored the pivotal role of lncRNAs in driving macrophage differentiation towards the M2 phenotype, typified by anti-inflammatory reactivity and pro-regenerative attributes, thereby manifesting tissue reparative potential. Moreover, lncRNAs might interact with the genome to control chromatin dynamics or act as a structural base for protein complexes involved in regulating macrophage polarization. Furthermore, exocrine lncRNAs have the potential to modulate macrophage polarization via paracrine mechanisms, wherein they act on neighboring cells in the microenvironment (39). Several research studies have indicated that dysregulation of lncRNAs is linked to diverse human disorders such as vascular diseases (40), sepsis (41), different types of cancer (42–44), neurological disorders (45), and respiratory ailments (46, 47). In particular, lncRNAs have been found to be associated with various respiratory conditions, including lung cancer, acute lung injury/acute respiratory distress syndrome, interstitial lung diseases, infectious pulmonary diseases (such as tuberculosis and pneumonia), and chronic airway ailments (such as asthma, chronic obstructive pulmonary disease, and cystic fibrosis) (46). lncRNAs have emerged as a promising avenue for investigating the etiology of ALI and exploring novel therapeutic targets. Numerous lncRNAs, including MALAT1, NEAT1, TUG1, THRIL, CASC2, SNHG5, PRNCR1, Hsp4, lncRNA-5657, CLMAT3, MEG3, CASC9, XIST, GAS5, SNHG14, H19, Mirt2, and others, have been identified as potential therapeutic targets for the treatment of ALI (38). **Table 1** provided a comprehensive summary of the various lncRNAs that had been implicated in regulating macrophage function during ALI/ARDS. Despite recent research on the involvement of lncRNAs in macrophage development and activation, the potential of lncRNAs as a therapeutic tool for regulating macrophage polarization and their potential value in treating ALI/ARDS require further investigation in future studies.

**Table 1:** LncRNAs involved in ALI/ARDS macrophage function.

lncRNAs	Reference	Model and or cell	Expression	Function	Molecular targets
lncRNA-5657	Liu et al. (48)	LPS-induced ALI mouse model, patients with sepsis-induced lung injury, NR8383 cell line	Increased	Blocked the proinflammatory function of lncRNA-5657 in alveolar macrophages	Spns2
MEG3	Liao et al. (49)	LPS-induced ALI mouse alveolar and macrophage NR8383 cells	Increased	Improves inflammatory response	MicroRNA-7b (miR-7b)/NLR pyrin domain containing 3 (NLRP3)
SNHG14	Zhu et al. (50)	LPS-induced ALI mouse model	Increased	Reduces the levels of pro-inflammatory cytokines and inhibits alveolar macrophages MH-S cell viability.	miR-34c-3p/WISP1
H19	Mu et al. (51)	LPS-induced ARDS in rats and alveolar macrophage cells (MH-S) cells	Increased	Relieved the pulmonary injury, inflammation and fibrosis	miR-423-5p/FOXA1
H19	Hao et al. (52)	Sepsis-induced ALI mouse model	Increased	Increased the number of macrophages in BALF, apoptosis and induced pro-inflammatory cytokine production	miR-107/TGFBR3
Mirt2	Du et al. (53)	LPS induced primary cultured peritoneal macrophages, RAW264.7 cells	Increased	Regulate macrophage polarization and prevent aberrant activation of inflammation	TRAF6, NF-κB, stat6 and MAPK
LINC01194	Shen et al. (54)	LPS-induced ALI mouse model	Increased	Promotes the inflammatory response and apoptosis of lipopolysaccharide-treated MLE-12 cells	miR-12a-203p/MIP-3
lincRNA-Cox2	Robinson et al. (55)	LPS-induced ALI mouse model	Increased	Regulate Inflammation in Alveolar Macrophages	None

### 3.2 Immunometabolism regulates macrophage polarization

**Figure 5:**
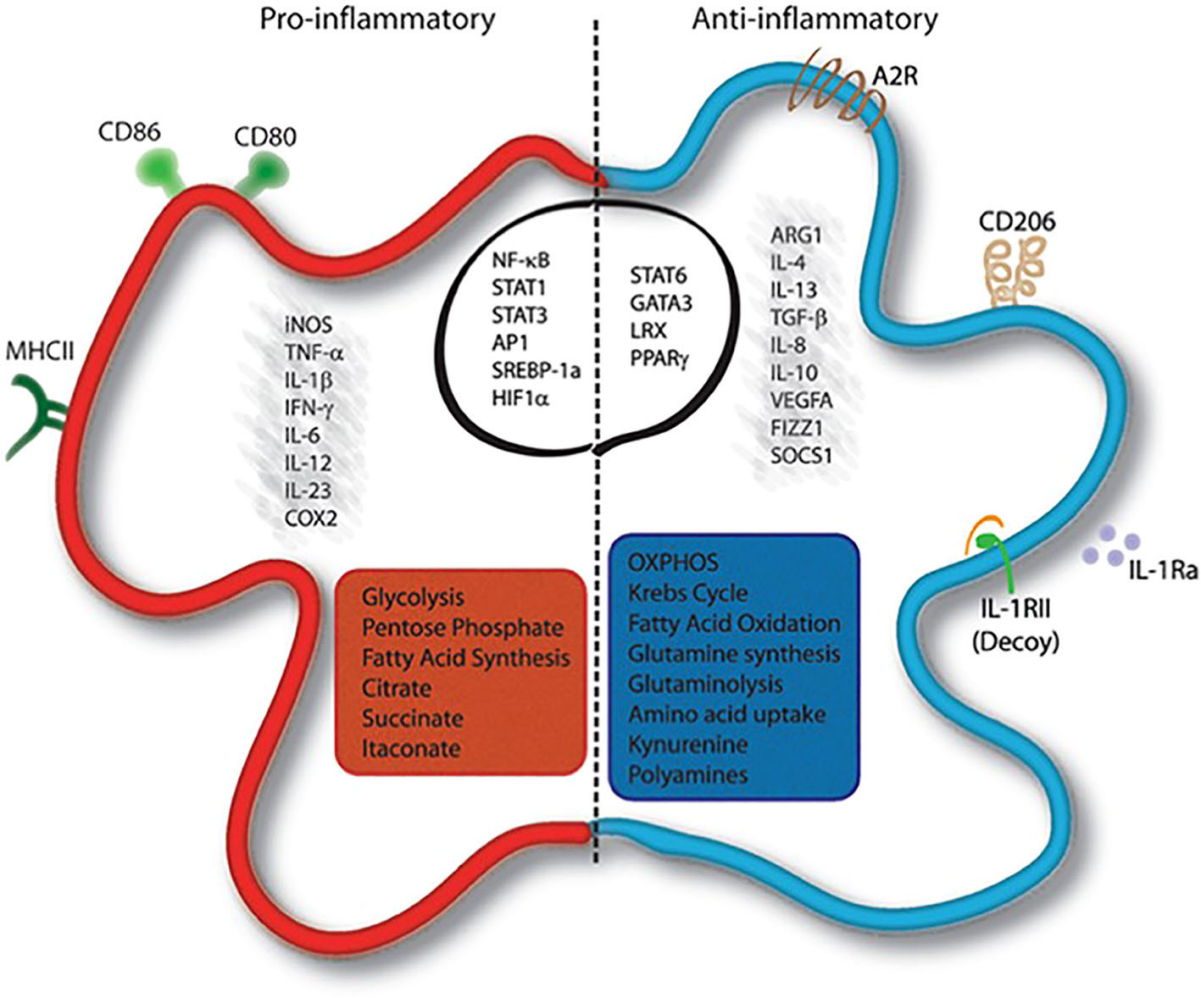
Activated macrophages exhibit distinct molecular and metabolic features (56).

In addition to the functional aspects discussed in **Figure 5**, metabolic reprogramming is also a critical component of macrophage polarization. Macrophages can dynamically shift from oxidative phosphorylation-based aerobic metabolism to glycolysis-based anaerobic metabolism, and vice versa, in response to various stimuli present in their microenvironment. Upon being exposed to pro-inflammatory stimuli, transcription factors become activated thereby instigating the production of molecules. Additionally, these macrophages undergo metabolic reprogramming, resulting in a shift towards the production of fatty acids, increased glycolysis, and enhanced activity of the pentose-phosphate pathway (56). Therefore, immunometabolism reprogramming plays a vital role in regulating macrophage function and is an essential aspect of their polarization.

Upon being exposed to pro-inflammatory stimuli, these macrophages undergo metabolic reprogramming, resulting in a shift towards the production of fatty acids, increased glycolysis, and enhanced activity of the pentose-phosphate pathway. Conversely, anti-inflammatory macrophages are characterized by cytokines. The precise modulation of these macrophage populations is orchestrated by transcriptional regulators such as signal transducer and activator of transcription 6 (STAT6), GATA binding protein 3 (GATA3), peroxisome proliferator-activated receptors (PPARs), and liver receptor homolog-1 (LRX) (56).

#### 3.2.1 Glycometabolism

The field of immune cell metabolism has a history tracing back to the 1950s, when the first set of studies emerged. These studies demonstrated that glycolysis was determined to be the primary functional mode of M1 type macrophages. In 1970, Hart and colleagues (57) observed that M1 macrophages, which were classically activated macrophages that responded to phagocytosis or inflammatory stimulation, displayed an enhanced rate of glycolysis and diminished oxygen consumption. These macrophages with pro-inflammatory characteristics predominantly utilize glycolysis and the pentose-phosphate pathway as the means to meet their adenosine triphosphate (ATP) requirements (56). Upon infection with gram-negative bacteria and subsequent activation of macrophages via LPS, significant metabolic changes occur, including upregulation of aerobic glycolysis and the pentose-phosphate pathway, as well as suppression of the tricarboxylic acid cycle. These metabolic pathways contribute to energy production by activated M1 macrophages facilitating cytokine production and antibacterial defense through Warburg metabolism (58). Overall, Metabolism is a vital component in the intricate regulation of macrophage function, exerting influence on the transcriptional and post-transcriptional machinery that governs the diverse functional states of these immune cells.

The glycometabolic pathway constitutes a fundamental mechanism in the regulation of the inflammatory response of M1 macrophages, exerting a multifaceted inhibitory effect on key cellular functions such as phagocytosis, reactive oxygen species (ROS) generation, and the secretion of pro-inflammatory cytokines (59–62). This regulatory capacity is mediated by the activation of diverse transcriptional regulatory elements, among which HIF1α assumes a critical role in promoting glycolysis even in normoxic scenarios. The transcription regulation of HIF1α is governed by a complex network of signaling pathways unique to macrophages, serving as an essential effector of innate immune response (56). Of the various signaling pathways implicated in the mechanism of inflammation, the Toll-like receptor/nuclear factor-κB (62) and AKT/mTOR (63, 64) pathways emerge as significant routes. Inflammatory stimuli are detected via a range of pattern recognition receptors, culminating in the initiation of a signaling cascade mediated by nuclear factor-κB. This pathway is a crucial regulator of macrophage function, which includes the transcriptional regulation of HIF1α-related genes. The AKT/mTOR pathway is activated in response to growth factors and pathogen sensing receptors. This signaling pathway facilitates the transcription of genes associated with mitochondrial biogenesis and oxidative metabolism. Notably, the Akt kinase appears to exert subtype-specific effects on macrophage polarization. Specifically, the absence of Akt1 promotes M1 polarization, whereas Akt2 deficiency induces M2 polarization (56).

There is ongoing discourse surrounding the significance of glycolytic flux in the mechanism of action of M2 macrophages. Numerous investigations have suggested that the glycolytic pathway is implicated in M2 macrophage polarization. However, divergent findings indicate that M2 polarization and its functional attributes can be compromised by hindering glycolysis through the use of 2-deoxyglucose, which is a potent inhibitor of glycolysis (65, 66). More recently, new data has emerged indicating that M2 differentiation might not require glycolytic flux as long as oxidative phosphorylation remained intact (67). Studies have proposed that M2 macrophages possess a more diverse metabolic activity profile. One of the key factors that regulate glycolytic activity in M2 macrophages is the selective expression of PFKFB1. This enzyme facilitates the efficient breakdown of the glycolytic stimulator fructose-2,6-diphosphate into fructose-6-phosphate, which results in a reduction in overall glycolysis rates. Moreover, it has been proposed that the increased expression of the carbohydrate kinase-like protein in M2 polarization helps enhance the non-oxidative branch of the pentose phosphate pathway, resulting in the generation of ribose-5-phosphate. Apart from its involvement in nucleotide synthesis, ribose-5p plays a crucial role in the synthesis of UDP-GlcNAC and N-glycosylation, which are significant processes for protein modification and cell signaling. N-glycosylation is essential for modifying various cell surface proteins, including CD206, which is widely expressed in M2 macrophages (56). The contribution of glycolysis to M2 macrophage polarization remains controversial, necessitating further investigation to delineate its context-specific regulatory mechanisms across alveolar macrophage polarization states in ALI/ARDS.

#### 3.2.2 Lipid metabolism

Macrophages are capable of phagocytosing various types of lipids through phagocytosis, scavenger receptor-mediated pathways, and macrophage action from dead cells and the surrounding environment. After the recognition of inflammation-induced signals, lipids present in the affected area are processed by acid lipase enzymes and instigates the hydrolysis of lipids into unbound fatty acids and cholesterol molecules. Said fatty acids are then transferred to the mitochondria, where they are subjected to oxidative degradation via the mechanism of fatty acid oxidation, concomitantly resulting in numerous byproducts that sustain the acetyl coenzyme A or electron transport chain cycle. The polarization of M1 and M2 macrophages is controlled by their reliance on fatty acid synthesis and oxidation pathways (68). M1 macrophages promote fatty acid synthesis in response to inflammatory signals while inhibiting M2 macrophage differentiation, which relies on fatty acid oxidation. The differential regulation of fatty acid synthesis and oxidation plays a crucial role in macrophage polarization towards M1 or M2 phenotypes (56).

It is noteworthy that distinct populations of tissue resident macrophages exhibit diversified transcriptional, epigenetic, and metabolic profiles. For instance, in comparison with other macrophage types, AMs display enriched functions related to transcription. Furthermore, several transcription factors (TFs) have been shown to be closely associated with specific auxiliary AMs functions. Lipid metabolism is regulated at the transcriptional level by a diverse array of transcription factors in AMs, including the peroxisome proliferator-activated family of receptors (PPARs) and the CCAAT enhancer binding proteins (C/EBPs), among others. These transcription factors play intricate and complex roles in regulating the expression of genes implicated in lipid metabolism, with their interplay contributing to the overall regulation of lipid metabolism (56, 68). PPARs are expressed by AMs, and they play a crucial role in regulating the development and maintenance of AMs as well as the polarization of M2 macrophages (56, 68). Despite the demonstrated anti-inflammatory effects of PPARγ in macrophages, the mechanisms underlying its expression *in vivo* and the means through which it regulates tissue homeostasis and inflammation remain poorly understood. Gautier et al. (69) demonstrated that PPARγ deficiency in pulmonary macrophages led to mild lung inflammation in a stable state. PPARγ has been recognized as a crucial mediator in the promotion of regression of inflammation, maintenance of pulmonary macrophage function, and bolstering host defense mechanisms in the lungs. Previous research conducted by Schneider et al. (70) outlined that granulocyte-macrophage colony stimulating factor on perinatal AMs in lung development PPARγ in fetal monocytes. The C/EBP family of TFs comprises basic leucine zipper (bZIP) proteins involved in the regulation of glucose and lipid metabolism, as well as M2 macrophage polarization. Notably, C/EBPβ has been implicated in the maintenance of AMs, as C/EBPβ^-^/^-^ mice reportedly lack these populations based on the absence of characteristic surface markers (68). Therefore, the targeted modulation of PPARγ or C/EBPβ may potentially offer a viable therapeutic strategy for ameliorating inflammation in individuals suffering from ALI and ARDS.

#### 3.2.3 Amino acid metabolism

The metabolic pathways of amino acids play a crucial role in coordinating immune responses and sustaining optimal immune functionality. Furthermore, macrophages, which are pivotal elements of the immune system, heavily rely on adequate levels of amino acids to facilitate their activation and immune-related functions adequately. Amino acid metabolism in macrophages is also known to regulate various macrophage functions, such as the mTOR signaling pathway and the production of nitric oxide. Any perturbations in amino acid metabolism can have immunomodulatory effects on macrophage responses, leading to altered immune functions. Current studies on macrophage amino acid metabolism have mainly concentrated on arginine, tryptophan, and glutamine, while investigations in alveolar macrophages remain scarce (56). Therefore, further investigation into amino acid metabolic pathways in alveolar macrophages is warranted, as this may offer a novel and promising therapeutic approach for ALI/ARDS.

## 4 Macrophage migration

During inflammatory conditions, inflammatory mononuclear cells undergo migration towards damaged tissues where they differentiate into tissue macrophages or dendritic cells (71). The regulation of AMs recruitment is critical for mitigating inflammation and protecting pulmonary tissue during ALI (72). Over the past few years, the research focus has shifted towards studying the migration patterns of AMs (73–75). Kulle et al. (73) demonstrated through proteomic analysis that exposure to berry-flavored vapor altered cytoskeletal functions in AMs, leading to reduced cellular motility. Jaffal et al. (74) reported that hyperoxemia compromised AMs migration, phagocytosis, and bactericidal activity during hyperoxic acute lung injury, resulting in prolonged mechanical ventilation. Tsai et al. (75) reported that lung fibroblasts secreted the chemokine CXCL10, which facilitated activation and migration of resident macrophages during acute lung injury. Therefore, the recruitment and subsequent activation of AMs play a crucial role in resolving the initial inflammatory response and facilitating the tissue healing progress following inflammation or injury. Therefore, further exploration into the intricate mechanisms underlying AMs migration during ALI/ARDS may provide a more comprehensive understanding of the ailment’s pathogenesis, enabling innovative therapeutic interventions.

## 5 Immune function of macrophages

AMs act as sentinel cells and are critical gatekeepers in the innate immunity of the respiratory tract (76). AMs are also highly differentiated cells that undergo self-renewal to maintain their population in stable conditions (77). Under normal circumstances, AMs serve as anti-inflammatory cells and prevent immune pathologies and specific immune responses to harmless antigens (28). During immune recognition of pathogen-related molecular patterns via PRRs, such as TLRs, NLRs, and C-type lectin receptors, AMs maintain their ability to recognize and respond to pathogens and other harmful signals, thereby initiating both innate and adaptive immune responses (28). Therefore, AMs ontogeny and inflammatory status govern compartmentalized regulation of innate immune networks. The innate immune system plays a pivotal role in protecting the host from invading pathogens through the recognition and response to diverse microbial and PAMPs. The untoward recognition of DAMPs can disrupt a delicate immune homeostasis, leading to the development of chronic inflammation and tissue damage if left unresolved (9). Consequently, the innate immune system has a pivotal role in ensuring a fine-tuned balance between immune defense and tissue homeostasis. PRRs, which encompass a diverse set of receptors such as TLRs, NLRs, RLRs, CLRs, and intracellular DNA sensors including the cGAS-STING signaling pathway and AIM2-like receptors, are critical in recognizing a range of molecular patterns including PAMPs and DAMPs (78, 79). The activation of PRRs can lead to the trigger of immune responses that involve the production of cytokines, chemokines, interferons, and reactive oxygen and nitrogen species (ROS and RNS), amongst other mediators. These immune responses are essential for the eradication of infections and the maintenance of immune homeostasis. However, congenital immune response disorders during infections may heighten pathogen loads due to low pathogen clearance efficiency or aggravate and irreparably damage organs in patients with infection, thereby exacerbating its severity (80, 81). Therefore, the precise regulation of the innate immune response is vital in effectively combating infections throughout both acute stages of illness.

AMs are integral to the regulation of non-specific immune defense mechanisms, including the phagocytosis of intruding pathogens, production of inflammatory mediators, and expression of pro-inflammatory cytokines (82). Under normal physiological conditions, AMs have low levels of inflammatory cytokine production and high phagocytic activity, which generally inhibit inflammation and adaptive immunity (83). AMs constitute secretion of a variety of cytokines and chemokines, which are essential for the initiation of immune responses. The cytokines and chemokines produced by M1-like AMs, including TNFα, NO, IL-1β, IL-6, IFN, and macrophage inflammatory protein (MIP)-1α, are responsible for activating other immune cells, recruiting immune cells to the site of infection or inflammation, and regulating the immune response in the lungs (28). M1 macrophages respond to microorganisms and Th1 pro-inflammatory cytokines, releasing inflammatory cytokines, enhancing bacterial killing ability, glycolysis, and recruiting immune cells into the lung parenchyma and alveoli (83). Then, the excessive production of these mediators may lead to the pathophysiology of inflammatory respiratory disorders, such as ARDS (28). Additionally, M2-like AMs play a role in reducing inflammation by releasing anti-inflammatory mediators and clearing apoptotic bodies through exocytosis (76). M2 macrophages, on the other hand, undergo oxidative metabolism induced by exposure to Th2 cytokines, leading to the release of anti-inflammatory cytokines, phagocytosis of apoptotic cells, and collagen deposition, which contribute to reduced inflammation and tissue repair (82). In summary, AMs play an essential role in the maintenance of tissue homeostasis, immune defense, surveillance of pathogens, clearance of surfactant and cell debris, identification of pathogens, initiation and resolution of lung inflammation, and tissue repair in response to damage (82, 83).

The respiratory system of mammals is equipped with various immune cells that coordinate to protect against infections and maintain homeostasis. Among these cells are macrophages, which are classified into AM and IM. Additionally, alveolar and bronchial epithelial cells (AECs and BECs) contribute to the innate immune response in the lung. Dendritic cells (DCs), natural killer cells (NK cells), and other innate lymphoid cells (ILCs), including ILC1, ILC2, and ILC3, also participate in the immune response of the lung. Furthermore, adaptive immune cells, such as T and B cells, play a crucial role in the adaptive immune response to lung infections. Neutrophilic infiltration of the lungs is known to occur in response to infections or pathological inflammation (9).

**Figure 6:**
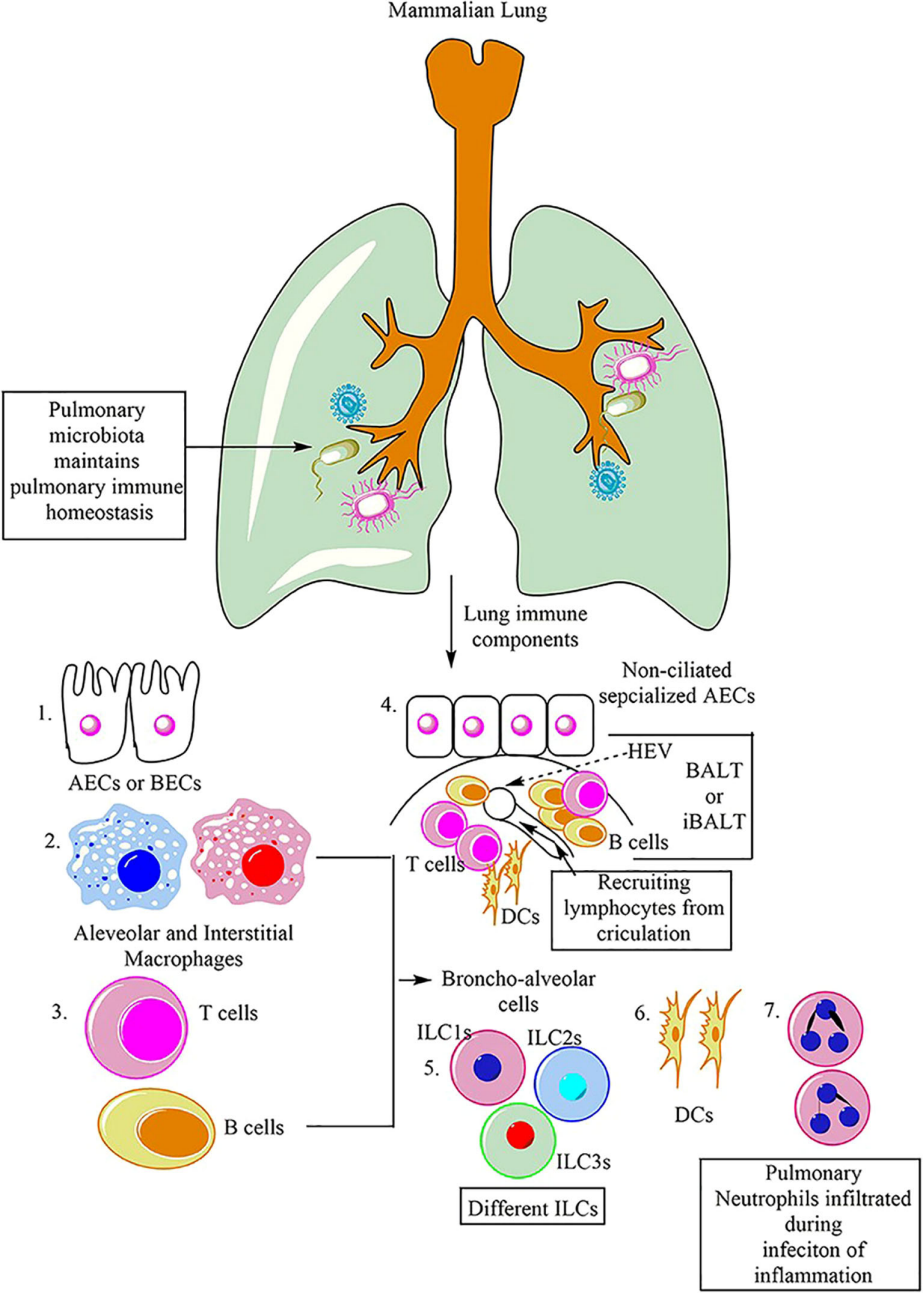
Main immune cells in mammalian lung (9).

**Figure 7:**
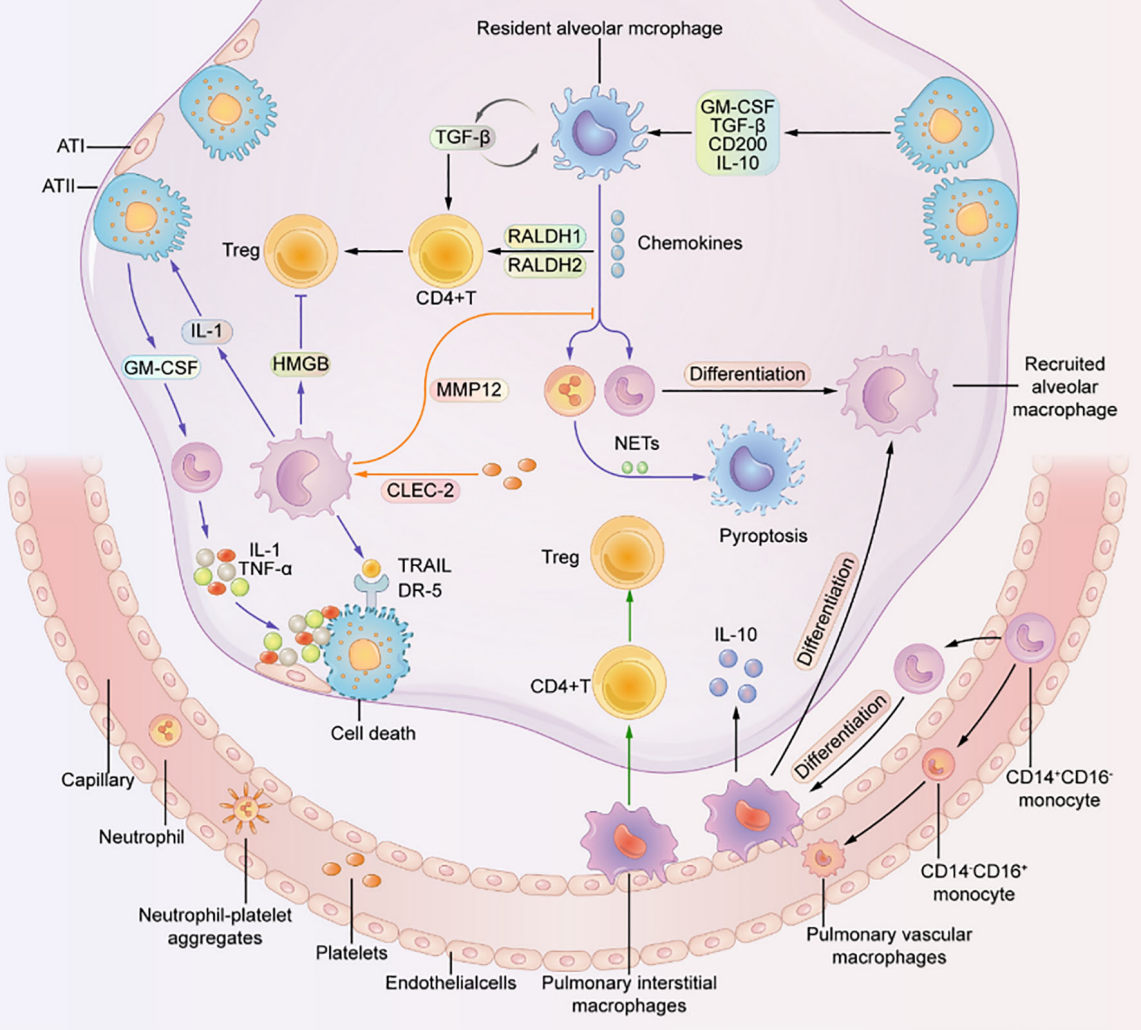
Macrophages residing in the pulmonary system are crucially important for the proper functioning of the lung (3).

ARDS is a severe inflammatory disorder marked by injury to alveolar epithelial cells, augmented alveolar-capillary permeability, and an accompanying inflammatory response (84). In the steady state, 90% of Broncho-alveolar cells (BACs) are alveolar macrophages, while 10% are lymphocytes (as shown in **Figure 6**) (9). Bronchoalveolar lavage fluid (BALF) is a biologically complex fluid that harbors a diversified assortment of innate immune cells including alveolar macrophages, innate lymphoid cells (ILCs), and dendritic cells (DCs), which function as essential antigen-presenting cells (APCs) in the lung (85). These innate immune cells are critical in preserving optimal respiratory health by detecting and eliminating various pathogenic agents, regulating inflammatory responses, and facilitating wound healing and tissue repair in the lungs (9). This is accomplished through intricate communication with various cell types and AMs, including epithelial cells, microvascular endothelial cells, neutrophils, lymphocytes, fibroblasts, and tissue progenitor cells (82). Activation of macrophages leads to the release of pro-inflammatory cytokines, recruitment of neutrophils and other leukocytes, the interaction with alveolar epithelial cells, cellular debris clearance, and the secretion of growth factors for regulation of inflammation and tissue remodeling. The remarkable characteristic of macrophages to adopt different phenotypes depending on the microenvironment is a pivotal attribute. TLRs expressed by RAMs and alveolar type II epithelial cells (ATII) detect PAMPs and trigger the secretion of chemokines, directing the migration of circulating immune cells such as monocytes, neutrophils, and platelets to the alveolar space (3). Immune cells release pro-inflammatory mediators that worsen the damage to alveolar epithelial and endothelial cells, causing an increase in vascular permeability and ultimately resulting in pulmonary edema (as shown in **Figure 7**) (3, 86, 87).

They interact with other cells to maintain a healthy state. Resident alveolar macrophages (RAMs) promote the differentiation of CD4 T cells into Treg cells. RAMs, interstitial macrophages, and recruited alveolar macrophages (RecAMs) respond to inflammation in different ways. Inflammation can cause RAMs to produce chemokines to attract other cells. RecAMs produce inflammatory factors that lead to apoptosis of alveolar epithelial cells and inhibit the differentiation of Treg cells. Platelet-expressed C-type lectin-like 2 (CLEC-2) interacts with RecAMs to hinder chemokine activity (3).

## 6 Cell death

Heterogeneous macrophage death mechanisms modulate the progression and severity of ALI-associated inflammation. As the primary and key leukocytes in the alveolar space, AMs can significantly influence the progression of ALI by synthesizing and releasing various inflammatory mediators in response to infectious and non-infectious stimulus. Recent scientific investigations have suggested that inflammation and cellular demise exhibit a synergistic effect, with each action enhancing the potency of the other. This, in turn, gives rise to an automatic amplification cascade that culminates in an unchecked inflammatory reaction (88). In biological systems, cells can undergo RCD through genetically encoded mechanisms. RCD is a crucial aspect of normal physiological programming, contributing to processes such as organ development and epithelial renewal. In addition, this phenomenon has been observed in cells that are unable to mitigate the pressure source that poses a threat to tissue homeostasis. Numerous physiological and pathological conditions can trigger cell death, including infection by intracellular pathogens or cellular dysfunction induced by DNA damage, oxidative stress, or protein misfolding. Until these recent discoveries, the scientific community had solely acknowledged two primary forms of cellular demise, specifically, apoptosis and necrosis. Despite the continued relevance of these morphological characteristics in comprehending RCD, there has been a growing diversification of forms of active cell death (89). Therefore, therapeutic interventions aimed at targeting the cellular mechanisms responsible for initiating signals that lead to the death of alveolar macrophages may represent a promising strategy for treating ALI/ARDS.

### 6.1 Apoptosis

During lung development and under homeostatic conditions, resident macrophages undergo apoptosis as part of physiological turnover. Similarly, during the resolution of inflammation, recruited macrophages also undergo programmed cell death. Animal studies indicate that both interstitial and AMs are susceptible to apoptosis; however, under steady-state conditions, interstitial macrophages exhibit a shorter lifespan and a higher apoptotic rate, potentially attributable to the continuous phagocytosis and degradation of surfactant proteins and innocuous particles by AMs. In the pulmonary environment, the intrinsic apoptotic pathway can be triggered by various stimuli, such as growth factor withdrawal, oxidative stress, endoplasmic reticulum stress, and DNA damage (89). In both *in vivo* and *in vitro* models of LPS-induced ALI, upregulation of the pro-apoptotic proteins Bax and caspase-3, alongside downregulation of the anti-apoptotic protein Bcl-2, indicates significant activation of apoptosis in AMs (7). Emerging evidence further underscores a link between the TLR4 signaling pathway and macrophage apoptosis. Jiang et al. (10) demonstrated that ligustrazine alleviated ALI by suppressing the TLR4/TRAF6/NF-kB/NLRP3/caspase-1 and TLR4/caspase-8/caspase-3 pathways, thereby reversing macrophage polarization and reducing both pyroptosis and apoptosis. In another study, Wei et al. (90) reported that elevated circ-Phkb expression suppressed AM proliferation via the TLR4/MyD88/NF-ĸB/CCL2 axis, promoting apoptosis and enhancing pro-inflammatory cytokine release. These findings suggest that modulation of TLR4 signaling may represent a promising therapeutic target for ALI. Additionally, activation of the Wnt/β-catenin pathway has been implicated as a key mechanism in LPS-induced apoptosis of AMs (91). A comprehensive understanding of the molecular regulation of death receptor signaling in AMs is therefore essential for developing targeted therapies to curb excessive cell death and improve clinical outcomes in ALI/ARDS. Collectively, these signaling networks maintain a delicate balance between AM survival and apoptosis in the lung. Disruption of this equilibrium underlies the pathogenesis of ALI/ARDS, highlighting the need for further mechanistic insights to guide effective treatment strategies.

### 6.2 Necrosis and necrotic apoptosis

**Figure 8:**
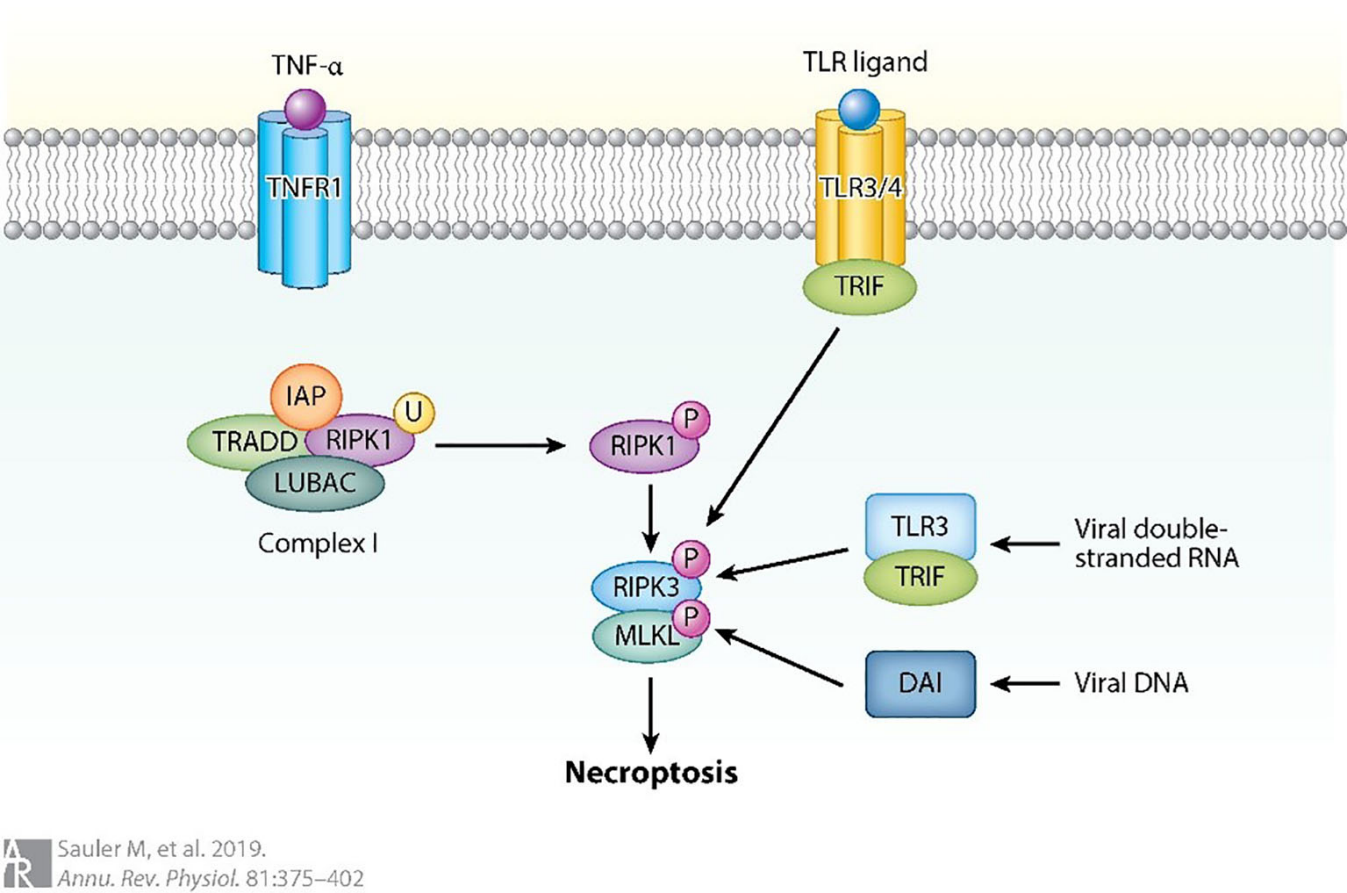
Necrotic apoptosis can also be triggered by the activation of other pathways (89).

Necrotic apoptosis, adhering to the morphological features of necrosis, represents a form of programmed cell death that has gained significant attention within the scientific community (as shown in **Figure 8**). TNFR1-mediated necrotic apoptosis is one type of this mechanism, where RIPK1 phosphorylates RIPK3 leading to the formation of a necrotic body, an amyloid signaling complex (89, 92). Previous studies have demonstrated that RIPK3-mediated necroptosis and inflammasome signaling are activated in LPS-induced ALI. Moreover, increased RIPK3 expression has been proposed as a potential biomarker for ventilator-associated ALI (7). This mechanism is facilitated by the participation of key molecular regulators, which support the proper execution of essential cellular functions. Once the necrotic body is generated, RIPK3 phosphorylates MLKL, which causes plasma membrane pores leading to cellular swelling and discharge of intracellular components like DAMPs (93). This process promotes inflammation as it releases pro-inflammatory mediators like HMGB1. Additionally, RIPK3 can promote inflammation through NF-κB, IL-1β, and IL-18 independent of pore formation during necrosis (89). In addition, a recent study revealed that AMs exhibited specific high-level expression of the leptin receptor (Lepr). Among tissue-resident macrophages, AMs also showed elevated expression of MLKL (7). In a mouse model of influenza virus-induced ALI, Wan et al. (94) demonstrated that Osteopontin knockdown markedly reduced phosphorylated MLKL levels in AMs, thereby attenuating necroptosis and ameliorating lung injury. However, MLKL activation may not always lead to necrosis, as ESCRT-III is involved in mediating cell resistance to necrotic cell death or delayed necrotic cell death by restoring membrane damage through the release of broken vesicles (95). This mechanism allows cells to produce appropriate cytokines to activate the immune system (89).

For instance, when caspase 8 is absent, RIPK1 phosphorylation occurs, followed by RIPK3 oligomerization and phosphorylation of MLKL. DNA-dependent activator (DAI) is another regulator that can activate RIPK3-mediated necrotic apoptosis by detecting viral DNA. Toll-like receptor-3 (TLR3) can detect viral DNA and induce the production of interferon through the connector containing TLR domain-β (TRIF). This process can activate RIPK1 and lead to necroptosis independent of RIPK3. Similarly, TLR4 can activate RIPK1 through TRIF in the absence of RIPK3, resulting in necroptosis. The ubiquitination of Receptor Interacting Protein Kinase 1 (RIPK1) is considerably more widespread in conditions where the Inhibitor of Apoptosis Proteins (IAPs) and the Linear Ubiquitin Chain Assembly Complex (LUBAC) are absent (89).

### 6.3 Pyroptosis

**Figure 9:**
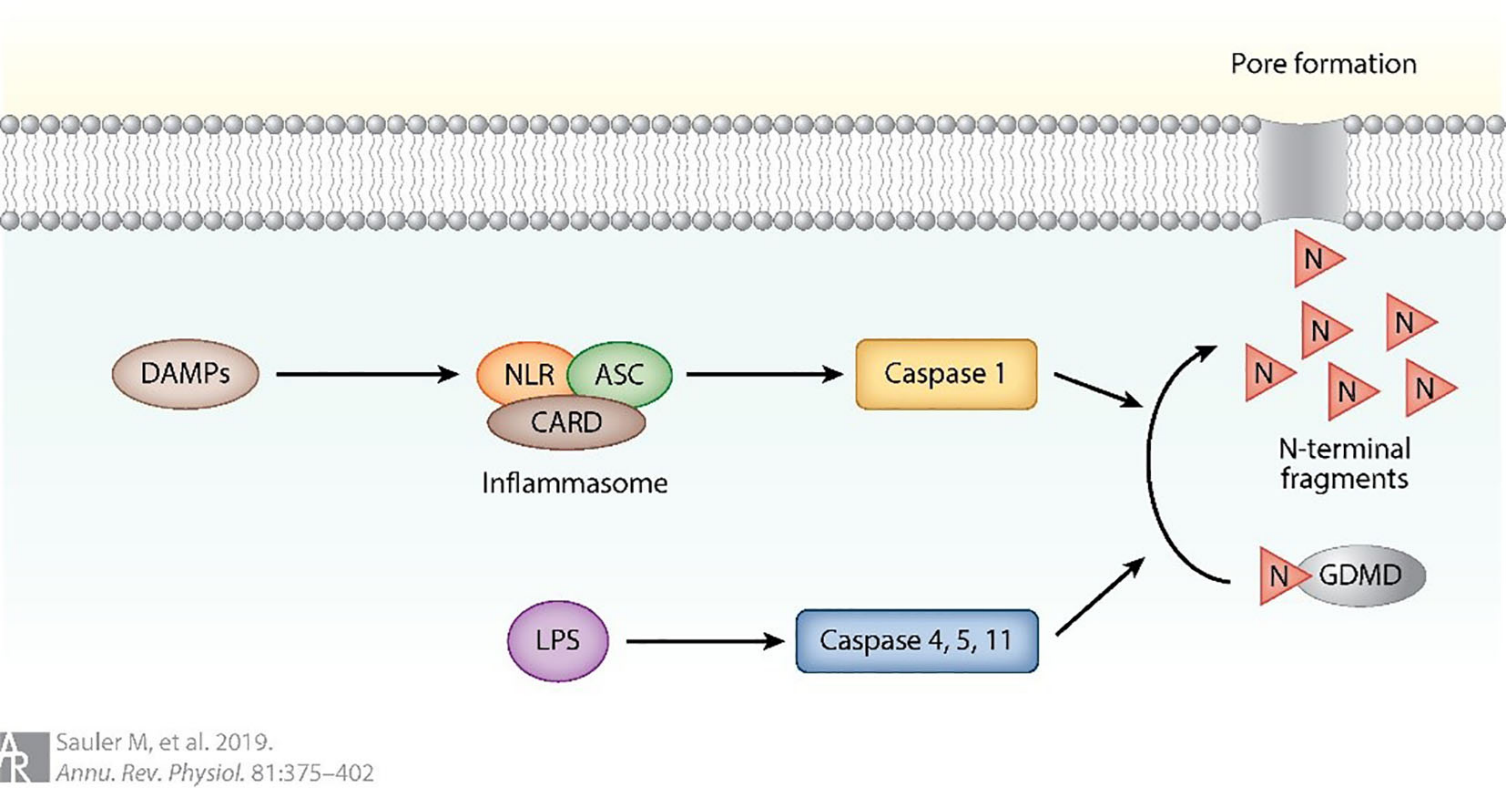
Pyroptosis can also be triggered by the activation of NOD-like receptors (89).

Pyroptosis is a form of programmed cell death that is essentially regulated by caspase-1 activation. Pyroptosis is initiated when NOD-like receptors (NLRs) are activated by DAMPs, leading to the formation of an inflammasome protein complex that includes apoptosis-related speck protein containing CARD (ASC) and caspase activation and recruitment domain (CARD). The activation of the inflammasome complex initiates a cascade of events, culminating in the cleavage of pro-caspase 1. This activated form of caspase 1 subsequently cleaves the N-terminal of Gasdermin D (GSDMD), generating N-terminal fragments that form proteinaceous pores in the cell membrane. These pores ultimately result in a form of regulated cell death, known as pyroptosis (as shown in **Figure 9**) (89). Upon activation, caspase-1 mediates the cleavage of GSDMD, resulting in mature GSDMD formation. Mature GSDMD is responsible for inducing cellular membrane pores, which consequently leads to cellular swelling, plasma membrane rupture, and the release of inflammatory cellular contents (88). The identification of gene deletion and pyroptosis that are dependent on GSDMD has greatly enhanced our understanding of the molecular mechanisms that control programmed necrotic cell death and inflammatory activation (96). Accumulating evidence indicates that pyroptosis plays a pivotal role in mediating the pulmonary inflammatory response during ALI. Targeting pyroptotic pathways has therefore emerged as a potential therapeutic strategy for ALI (11, 97–101). Pyroptosis is characterized by cellular swelling and plasma membrane rupture, resulting in the release of proinflammatory cytokines such as interleukin-1β (IL-1β) and interleukin-18 (IL-18) (102). Notably, pyroptosis of AMs following intratracheal LPS challenge markedly exacerbates pulmonary inflammation (7). Recent studies have highlighted several molecular and cellular mechanisms underlying the regulation of macrophage pyroptosis in ALI. Liu et al. (11) reported that mesenchymal stem cell-derived exosomes (MSCs-Exo) effectively alleviate ALI by suppressing AM pyroptosis and attenuating inflammation through the delivery of pyroptosis-targeting microRNAs and immunomodulatory proteins. Xu et al. (97) demonstrated that carbon monoxide mitigated LPS-induced ALI by inhibiting AM pyroptosis. Similarly, Han et al. (98) showed that irisin ameliorated ALI by suppressing the HSP90/NLRP3/caspase-1/GSDMD signaling cascade, reversing macrophage polarization, and reducing macrophage pyroptosis. In contrast, Jia et al. (99) revealed that USP48 promoted NLRP3-dependent pyroptosis of AMs, thereby aggravating sepsis-induced ALI, whereas their subsequent work (100) identified ULK1 as a negative regulator of this process, diminishing NLRP3 expression and restraining macrophage-driven immune activation. Moreover, Liu et al. (101) reported that mitophagy promoted demethylation of the miR-138-5p promoter, thereby suppressing NLRP3 inflammasome activation, alveolar macrophage pyroptosis, and inflammatory responses in sepsis-induced lung injury. Collectively, these findings underscore the central role of AM pyroptosis in ALI pathogenesis and highlight multiple potential therapeutic targets for intervention.

Pyroptosis is initiated upon activation of NOD-like receptors (NLRs) by damage-associated molecular patterns (DAMPs). This activation results in the assembly of an inflammasome protein complex comprising apoptosis-associated speck-like protein containing a CARD (ASC) and caspase activation and recruitment domain (CARD). Subsequent activation of the inflammasome complex triggers a cascade of events culminating in the cleavage of pro-caspase-1 (89).

### 6.4 Effect of ferroptosis on macrophages

**Figure 10:**
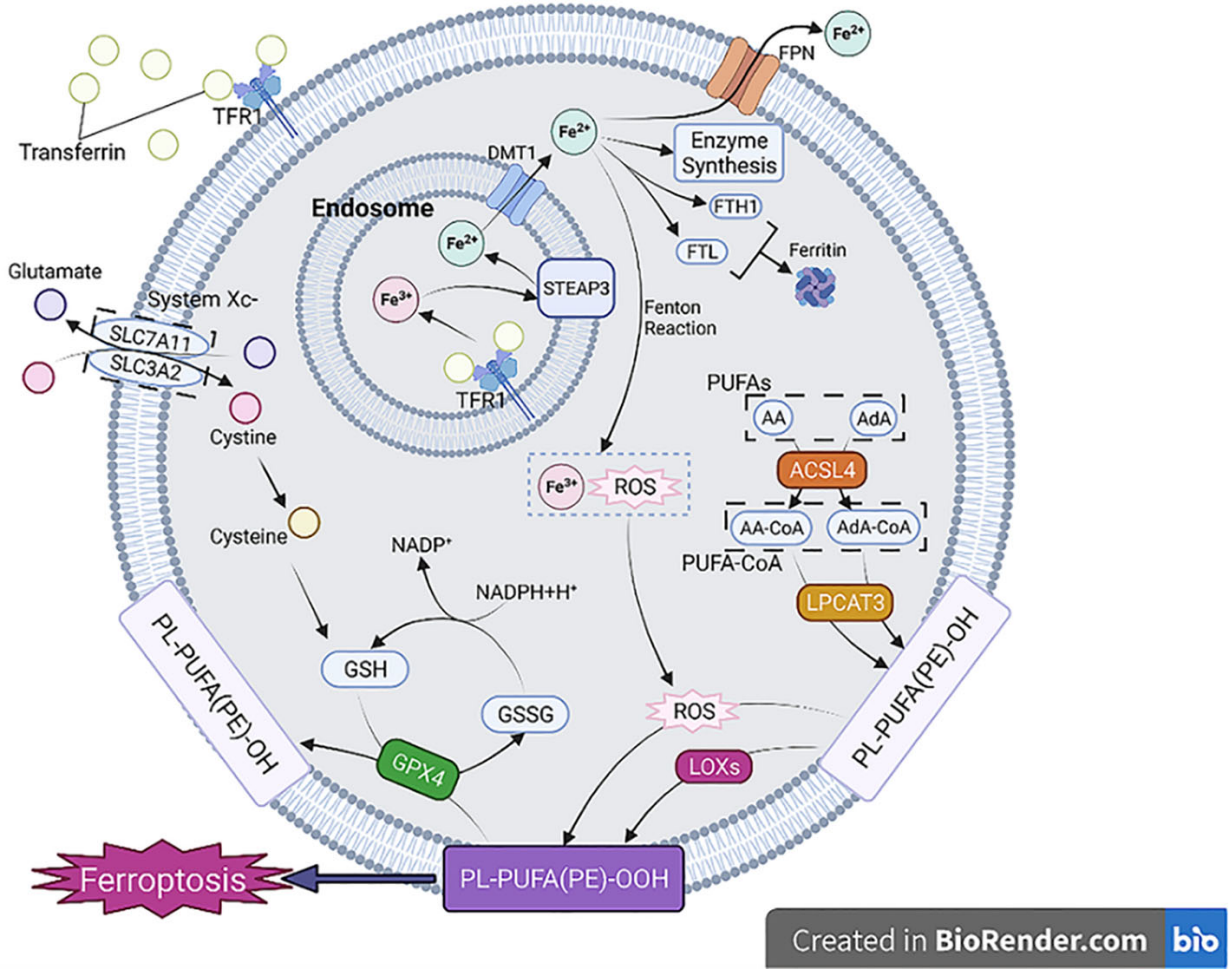
The process and classification of Ferroptosis (103).

Iron is indispensable for several physiological functions, such as cell differentiation, proliferation, and metabolism. The principal source of iron required for these functions comes from the recycling of iron from red blood cells by macrophages through complex mechanisms. The accretion of iron in macrophages can significantly alter their fate and function, especially during cell development and differentiation. Furthermore, iron accumulation within macrophages can trigger the production of ROS and lipid peroxidation through the Fenton reaction, leading to Ferroptosis. Furthermore, iron buildup in resident macrophages can have a ripple effect on other cells in various tissues. The hepcidin/ferroportin regulatory system typically maintains the body’s iron balance. However, if iron accumulation in macrophages disrupts this balance, uncontrolled iron output may cause tissue and systemic iron overload, creating an environment for Ferroptosis of tissue (103). In oxidative stress scenarios, the interplay between Fe^2+^ and Fe^3+^ plays a crucial role in the production of ROS. Ferroptosis, a form of regulated cell death, is associated with intricate regulation of ROS levels that is closely intertwined with genes involved in iron metabolism. Notably, several iron metabolism genes, including transferrin (TRF), Ferroportin, ferritin heavy chain (FTH), and ferritin light chain (FTL), contribute to the modulation of ROS levels by facilitating the conversion between Fe^2+^ and Fe^3+^. GPX4 and GSH, both governed by the Nrf2 pathway, are responsible for mitigating the detrimental effects of ROS. However, the depletion of these substances can lead to an accumulation of ROS in cells, resulting in lipid peroxidation. Furthermore, the heightened elicitation of ACSL4 and LOX induces the conversion of polyunsaturated fatty acids (PUFAs) into lipid peroxides, ultimately bolstering the occurrence of ferroptosis, as shown in **Figure 10**. The release of iron overload and Ferroptosis-related substances also impacts macrophage polarization and recruitment, revealing a complex interplay between Ferroptosis and the immune response (103). The effects of iron overload on macrophage polarization have been found to exhibit variation contingent upon the duration of the overload. Acute iron overload has the potential to induce M1 polarization, accentuating the expression of M1 markers including IL-6, TNF-α, and IL-1β, while concurrently reducing the expression of M2 markers such as transglutaminase 2 (TGM2) (104). Furthermore, iron overload-induced ROS production and p53 acetylation contribute to M1 polarization (105). In chronic iron overload, macrophages derived from THP-1 monocytes often exhibit M2 polarization, leading to the down-regulation of M1 macrophage markers (106). Ferroptosis and iron overload additionally affect enzyme activity involved in iron, lipid, and amino acid metabolism (103). The expression of iron-related genes shifts depending on the stage of macrophage polarization, with M1 macrophages exhibiting higher levels of Hamp and FTH/FTL but lower levels of FPN and IRP1/2 (107). During Ferroptosis, iron death cells release HMGB1, inducing inflammation and macrophage recruitment, by activating molecular inflammatory pathways. Furthermore, Ferroptosis triggers the expression of various inflammation-related genes, including CCL2 and CCL7, which have vital roles in the chemotaxis and recruitment of macrophages (103). HMGB1 required specific receptors for advanced glycation end producted to mediate macrophage inflammation. More importantly, the consumption of anti-HMGB1 neutralizing antibody or AGRE could alleviate the inflammatory response of macrophages, which suggested that limiting the expression of HMGB1 might be a method to deal with macrophage inflammation (108). Apart from HMGB1, ferroptosis also induces inflammation and macrophage recruitment by activating molecular inflammatory pathways. Specifically, ferroptosis triggers the expression of various inflammation-related genes, particularly CCL2 and CCL7, which play pivotal roles in the recruitment and chemotaxis of macrophages (103). Ferroptosis induced the expression of various inflammation-related genes, especially CCL2 and CCL7, which contributed to the recruitment and chemotaxis of macrophages (103). Ferroptosis has been implicated in the pathogenesis of various diseases, including but not limited to nervous system diseases (105, 109, 110), cancer (111–113), ischemia-reperfusion injury (114, 115), and multi-organ dysfunction induced by sepsis (such as heart injury, ALI, liver injury, and acute kidney injury) (116).

RCD pathways, particularly ferroptosis—an iron-dependent process characterized by excessive lipid peroxidation and oxidative stress—have been implicated as key contributors to the progression of RDS (117). Recent scientific inquiries have demonstrated the crucial involvement of ferroptosis in ALI and ARDS, thereby highlighting the potential of ferroptosis inhibition as a promising therapeutic avenue to ameliorate ALI (114, 118–122). For instance, somatostatin-1 has been observed to rescue the downregulation of ferroptotic markers in LPS-induced ALI, while diencephalic astrocyte-derived neurotrophic factor (MANF) has been shown to alleviate LPS-induced ALI (12). Similarly, other investigations have linked ferroptosis to ALI pathogenesis in scenarios such as ischemia-reperfusion injury, radiation-induced lung injury, and seawater drowning (118–120). In addition, ferroptosis of AMs has also emerged as a crucial contributor to the pathogenesis of ALI/ARDS, and targeting this pathway may have therapeutic implications for the management of these conditions. Ye et al. (123) reported that miR-223-3p contained in LPS-induced extracellular vesicles (LPS-EVs) contributed to sepsisassociated ALI by priming AMs for autophagy and ferroptosis via the MEF2C/Hippo signaling pathway. Similarly, Wang et al. (121) demonstrated that AM-derived exosomal tRF-22-8BWS7K092 activated the Hippo signaling cascade to induce ferroptosis during ALI. In addition, Liang et al. (124) revealed that the RNA methyltransferase ZC3H13 enhanced m^6A methylation of PRDX6 mRNA in a YTHDF2-dependent manner, thereby modulating PRDX6 expression, regulating the p53/SLC7A11 axis, and promoting ferroptosis in AMs, which ultimately exacerbated sepsis-induced ALI. Alveolar macrophage-derived exosomal has been found to activate the Hippo signaling pathway, leading to ferroptosis in ALI (121). Discussion of its therapeutic implications for macrophage ferroptosis needed to validate clinical applications in ALI/ARDS in the future.

Ferroptosis is a regulated cell death process characterized by the accumulation of iron-dependent lipid peroxides in cell membranes, culminating in membrane damage and ultimately cell death. The ferroptotic phenomenon is intricately linked to three distinct metabolic routes, namely, iron metabolism, lipid metabolism, and amino acid metabolism. Iron metabolism plays a critical role in the initiation of ferroptosis (103).

### 6.5 Autophagy

**Figure 11:**
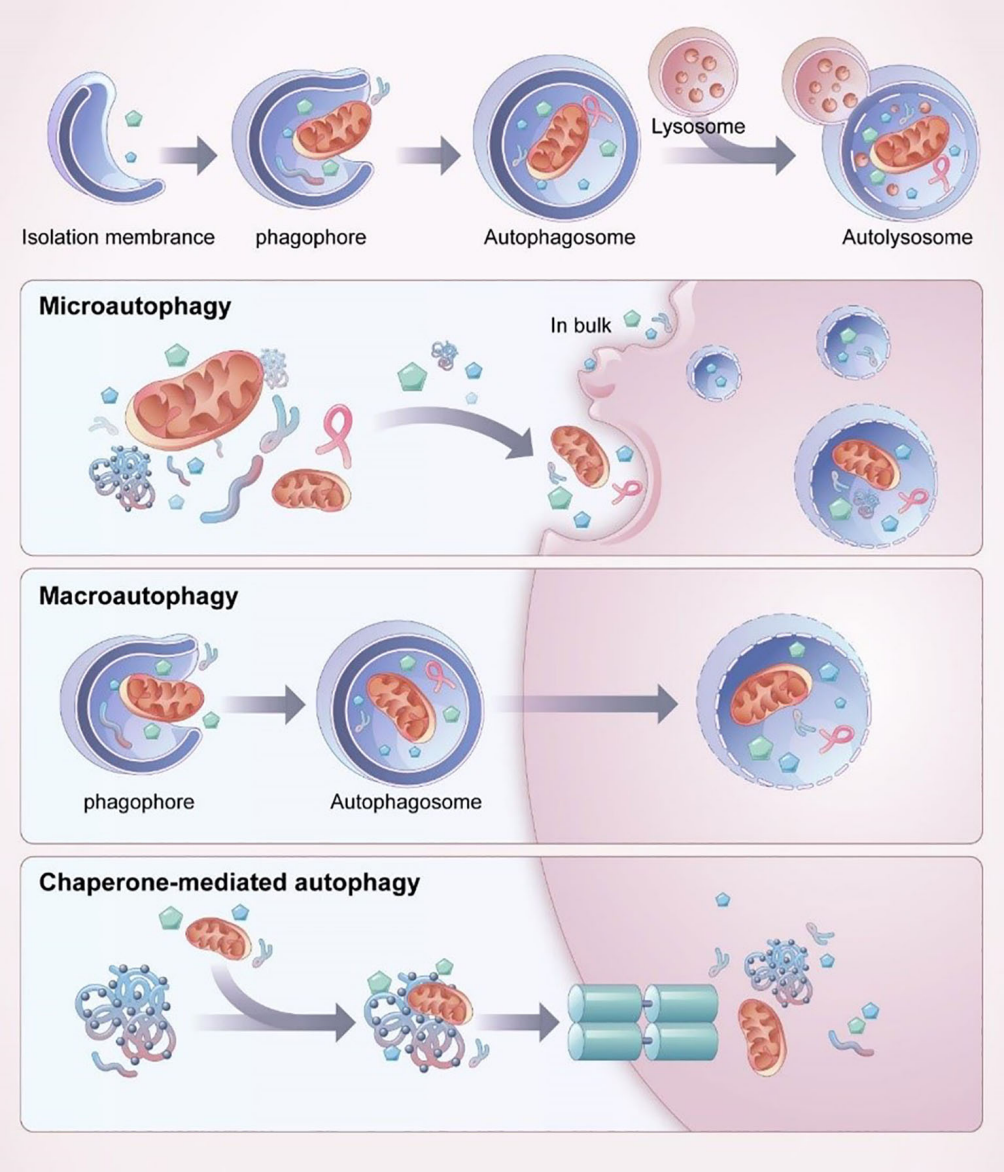
The process and classification of autophagy (12).

Disruption of autophagy flux following ALI has been shown to contribute to poor outcomes associated with autophagy dysfunction (13, 125). Autophagy is a crucial mechanism that facilitates cells to adjust to external environmental variations, uphold internal environmental stability, and combat foreign pathogen invasions. Within the domain of autophagy research, there are presently three primary forms of autophagy that are widely recognized, these being chaperone-mediated autophagy (CMA), microautophagy, and macroautophagy. Among these types, macroautophagy is the most dominant form, as shown in **Figure 11** (12). In all three forms, damaged organelles or proteins thereby contributingasis. Autophagy is a fundamental cellular mechanism that encompasses a series of coordinated processes, classified into distinct types based on the distinct modes by which intracellular components are transported to lysosomes for degradation and recycling. Macroautophagy involves the formation of membranes from the endoplasmic reticulum, golgi apparatus, or cytoplasmic membrane, which wrap around the material to be degraded, forming an autophagosome that subsequently fuses with lysosomes to degrade its contents. In contrast, microautophagy is formed when the lysosomal membrane directly engulfs long-lived proteins, among other cellular components, and degrades them within the lysosome. CMA, on the other hand, involves the binding of cytoplasmic proteins to molecular chaperones, which are then transported to the lysosomal lumen for degradation by lysosomal enzymes. CMA selectively targets soluble protein molecules for degradation, exhibiting some degree of selectivity, but not as much as in other forms of autophagy (126). Current research has prioritized investigating the significance of AM autophagy in the pathogenesis of ALI (12, 125, 127–129). The effects of autophagy on ALI/ARDS are variable and may be either protective or injurious, depending on the physiological context (7). The impact of autophagy on this condition can differ depending on the extent of lung injury and the equilibrium between pro-inflammatory cellular death and anti-inflammatory factors (13, 125).

In microautophagy, the cytoplasmic material to be degraded is directly engulfed by the lysosomal membrane, which invaginates or protrudes to engulf the substrate. In the physiological process of chaperone-mediated autophagy, molecular chaperones with substrate selectivity recognize and bind to specific motifs present in target proteins, facilitating their transportation to lysosomes for subsequent degradation. In macroautophagy, the substrate is engulfed by a double-membrane structure called the autophagosome, which then fuses with the lysosome to degrade and recycle the content (12).

The autophagic process in macrophages plays a pivotal role in mitigating pulmonary inflammation and injury (12). This protective effect is largely mediated through the suppression of NLRP3 inflammasome activation. The NLRP3 inflammasome, a cytoplasmic multiprotein complex, serves as a crucial sensor of pathogenic and stress signals, promoting the maturation and release of pro-inflammatory cytokines such as IL-1β and IL-18, thereby contributing to the pathogenesis of diverse inflammatory disorders (130, 131). By restraining the assembly and activation of NLRP3 inflammasomes, macrophage autophagy effectively attenuates lung inflammation and tissue damage. Regulation of autophagy in AMs is intricately linked to the MAPK signaling pathway, which orchestrates key cellular processes including proliferation, differentiation, and apoptosis. Dysregulation of MAPK signaling has been implicated in the development of ALI and ARDS (12). Targeting this pathway has thus emerged as a promising therapeutic approach. Liu et al. (132) demonstrated that BML-111, an agonist of the lipoxin A4 receptor, alleviated ALI by inducing AM autophagy through selective inhibition of the MAPK1 and MAPK8 pathways. The mTOR pathway also critically regulates autophagic responses. Activation of mTOR signaling promotes inflammation and inhibits autophagy, as evidenced by increased expression of p-mTOR, p62, and Beclin-1, a reduced LC3-II/LC3-I ratio, and elevated inflammatory mediator levels following LPS exposure in ALI models (132). Conversely, treatment with GYY4137, a novel H_2_S donor, reversed these alterations by inhibiting mTOR activation, thereby restoring autophagic balance and ameliorating lung injury. MicroRNAs further modulate autophagic signaling in AMs. For instance, microRNA-384-5p (miR-384-5p), delivered via bone marrow mesenchymal stem cell-derived exosomes, has been shown to regulate autophagy-associated signaling, leading to reduced expression of Beclin-1, a key regulator of autophagy initiation (13, 127). The functional role of Beclin-1 is determined by its interactions with specific autophagy inducers and repressors, influencing the dynamics of the autophagic process. Additionally, Wu et al. (128) demonstrated that Sesn2 deficiency suppresses LPS-induced mitophagy, resulting in increased ROS accumulation, mitochondrial damage, and pyroptosis in AMs. These findings suggest that Sesn2 confers protection against ALI by promoting mitochondrial autophagy and negatively regulating NLRP3 inflammasome activation. Collectively, these studies highlight macrophage autophagy as a central regulatory mechanism in ALI/ARDS pathogenesis. Therapeutic strategies that modulate autophagy—through MAPK or mTOR signaling, microRNA and so on-mediated regulation, or enhancement of mitochondrial autophagy—represent promising avenues for restoring pulmonary homeostasis and mitigating inflammation in acute lung injury.

Nonetheless, it should be noted that the impact of autophagy is not invariably beneficial, as evidenced by certain animal models in which macrophage autophagy was shown to exacerbate lung injury (12). For instance, both *in vitro* and *in vivo* experiments have demonstrated that in the presence of IgG immune complexes (IgGIC), complement components C5a and the membrane attack complex (MAC) synergistically promote the release of CXC and CC chemokines, thereby enhancing neutrophil recruitment and aggravating pulmonary damage (133). Sun et al. (133) further revealed that during acute lung injury, C5a induced apoptosis of AMs by binding to the C5a receptor and downregulating Bcl-2 expression. In a murine model of intestinal ischemia/reperfusion-induced ALI, activation of AMs through C5a–C5aR signaling triggered autophagy and apoptosis, with excessive activation leading to dysregulated LC3-II expression and disruption of pulmonary homeostasis. Pharmacologic inhibition of autophagy with 3-methyladenine (3-MA) or genetic silencing of ATG5 effectively reduced macrophage apoptosis and alleviated lung injury (134), suggesting that C5a-mediated autophagy activation may exacerbate ALI by promoting macrophage apoptosis.Temporal analyses in lipopolysaccharide (LPS)-induced ALI indicate distinct kinetics of cell death, with autophagy peaking at 2 hours and apoptosis occurring later at around 6 hours (135). Yang et al. (135) observed elevated macrophage autophagy and apoptosis in LPS-treated mice compared to controls, while resveratrol—a SIRT1 activator—suppressed both autophagy and apoptosis, concomitantly downregulating C5aR expression. Similarly, Qiu et al. (136) reported that excessive autophagy in AMs promoted apoptosis in LPS-induced lung injury, whereas hydrogen-rich saline alleviated inflammation by modulating macrophage polarization and inhibiting autophagy. In addition, recent findings indicate that the adaptor protein TRAF6 amplifies inflammatory signaling via NF-κB and MAPK activation (132); excessive autophagy enhances TRAF6 ubiquitination, further exacerbating lung injury. Collectively, these studies illustrate that macrophage autophagy can exert either protective or deleterious effects depending on its magnitude, timing, and upstream signaling context. At present, clinical investigations targeting autophagy modulation in ARDS remain limited. Further preclinical studies are required to delineate the optimal regulatory window for autophagy control, establish its long-term safety and efficacy, and develop precise therapeutic regimens for mitigating inflammation-associated lung injury.

Newborns, particularly preterm infants, represent a uniquely vulnerable population due to the structural and functional immaturity of their lungs. Even mild injury during this critical developmental window can profoundly impair pulmonary growth and function. In neonatal lungs, exposure to prenatal and early postnatal insults—such as infection, inflammation, and oxygen toxicity—converges to drive acute and chronic lung injury, ultimately manifesting as bronchopulmonary dysplasia. These pathological processes are characterized by excessive cytokine release, heightened protease activity, and persistent infiltration of innate immune cells, notably neutrophils and monocyte-derived macrophages (137). Recent neonatal studies employing animal models have demonstrated that restoration of autophagic flux can mitigate hyperoxia-induced lung injury (138–141). Mechanistically, this protection is mediated through several pathways, including calcitonin gene-related peptide (CGRP) signaling (138), modulation of the AMPK/mTOR/p53 axis (139), and RPTOR-dependent mechanisms (140). Conversely, Chen et al. (141) reported that activation of the TLR9–MyD88 signaling cascade enhanced NF-κB transcriptional activity, promoting pro-inflammatory cytokine production and contributing to ventilator-induced lung injury in neonates. Despite these advances, the spatiotemporal characteristics and mechanistic roles of AMs autophagy in neonatal ALI/ARDS remain poorly defined. Future research should focus on elucidating the functional dynamics of AM autophagy as both a biomarker and therapeutic target, and on clarifying its pathophysiological relevance across distinct stages and severities of neonatal lung injury.

## 7 Prospect

ALI falls under the clinical spectrum of ARDS. ARDS affects over 190,000 individuals annually in the United States, with mortality rates ranging from 26% to 58%. Despite improvements in supportive care, no pathophysiology-based therapies are currently available for ARDS. Beyond lung-protective ventilation and extracorporeal membrane oxygenation (ECMO)—which primarily support gas exchange and mitigate secondary injury—there remain no pathogenesis-targeted treatments, highlighting a significant unmet clinical need (3, 142). AMs play a central role in driving inflammatory responses and contribute substantially to the pathogenesis of ARDS. Their sequestration and infiltration into lung tissue impair gas exchange and worsen alveolar-capillary membrane disruption. Thus, anti-inflammatory strategies aimed at limiting AMs activation and tissue accumulation at inflammatory sites may help reduce associated morbidity and mortality.

The immunomodulatory potential of selective cytopheretic device (SCD) in ARDS was preliminarily investigated in a porcine model of acid-induced ALI (Humes HD, Buffington DA, Transportable renal replacement therapy for battlefield applications DoD/TARTRC, 2011–2013 [proposal application]). Briefly, anesthetized and mechanically ventilated pigs received 0.4N HCl via a tracheal catheter. SCD_Rx_ treatment in these animals resulted in reduced pulmonary vascular resistance and decreased leukocyte infiltration into lung tissue. Moreover, in a related congestive heart failure model, SCD_Rx_ promoted a shift in macrophage polarization—from a pro-inflammatory M1 phenotype observed in untreated dogs to a reparative/anti-inflammatory M2 phenotype in SCDRx-treated dogs. The modulation of peritoneal macrophages further indicated that SCD_Rx_ exerts not only organ-specific effects but also systemic immunomodulation (142). These findings suggest that SCD_Rx_ may alleviate alveolar inflammation and encourage M2 macrophage polarization, supporting its potential as a promising therapeutic strategy for ALI/ARDS.

The pathogenesis of ALI/ARDS is intimately linked to the functional imbalance of AMs—key orchestrators of pulmonary inflammation and tissue injury. Emerging interventional approaches targeting macrophage-related processes, such as epigenetic reprogramming, metabolic pathways, migratory behavior, and immune regulatory functions, as well as various forms of programmed cell death, have demonstrated potential in attenuating uncontrolled inflammation and reducing mortality in pre-clinical models. Nevertheless, the detailed mechanisms by which macrophage-driven metabolic shifts—specifically in glucose, lipid, and amino acid metabolism—integrate with apoptotic, pyroptotic, ferroptotic, and autophagic signaling pathways to influence ALI/ARDS progression remain inadequately defined. A deeper understanding of macrophage phenotypic heterogeneity and functional plasticity in the alveolar microenvironment is therefore essential to elucidate their context-dependent roles in lung injury and repair. Such insights will be critical for establishing a mechanistic basis upon which novel, target-specific clinical therapies can be developed.

## 8 Conclusion

The growing recognition among clinicians highlights the pivotal role of AMs in the response to lung injury, particularly the detrimental consequences of excessive and dysregulated inflammation in ALI/ARDS, and its subsequent impact on pulmonary function. This review has explored key mechanisms—including epigenetic and metabolic regulation, cell migration, immune modulation, and programmed cell death processes in AMs—to elucidate pathogenic pathways and potential immunomodulatory strategies for ALI/ARDS treatment. Nevertheless, the precise molecular mechanisms governing macrophage behavior, particularly their glycometabolic, lipid metabolic, and amino acid metabolic reprogramming, as well as their involvement in apoptosis, pyroptosis, ferroptosis, and autophagy, remain incompletely defined. Moreover, robust clinical and preclinical evidence supporting targeted immunomodulatory interventions remains limited. Therefore, further dedicated research is essential to clarify the phenotypic diversity and functional contributions of AMs in ALI/ARDS pathophysiology, which will ultimately facilitate the development of precise and effective therapeutic approaches.
